# Sorghum *Dw2* Encodes a Protein Kinase Regulator of Stem Internode Length

**DOI:** 10.1038/s41598-017-04609-5

**Published:** 2017-07-04

**Authors:** Josie L. Hilley, Brock D. Weers, Sandra K. Truong, Ryan F. McCormick, Ashley J. Mattison, Brian A. McKinley, Daryl T. Morishige, John E. Mullet

**Affiliations:** 1 Interdisciplinary Program in Genetics, Texas A&M University, 300 Olsen Boulevard, College Station, TX 77843 USA; 2 Department of Biochemistry and Biophysics, Texas A&M University, 300 Olsen Boulevard, College Station, TX 77843 USA

## Abstract

Sorghum is an important C4 grass crop grown for grain, forage, sugar, and bioenergy production. While tall, late flowering landraces are commonly grown in Africa, short early flowering varieties were selected in US grain sorghum breeding programs to reduce lodging and to facilitate machine harvesting. Four loci have been identified that affect stem length (*Dw1*-*Dw4*). Subsequent research showed that *Dw3* encodes an ABCB1 auxin transporter and *Dw1* encodes a highly conserved protein involved in the regulation of cell proliferation. In this study, *Dw2* was identified by fine-mapping and further confirmed by sequencing the *Dw2* alleles in Dwarf Yellow Milo and Double Dwarf Yellow Milo, the progenitor genotypes where the recessive allele of *dw2* originated. The *Dw2* locus was determined to correspond to Sobic.006G067700, a gene that encodes a protein kinase that is homologous to KIPK, a member of the AGCVIII subgroup of the AGC protein kinase family in Arabidopsis.

## Introduction

Sorghum is the fifth most widely grown cereal crop worldwide (faostat.fao.org). Its drought and heat tolerance make this crop especially important in semi-arid regions. Sorghum is a C4 grass with a diverse germplasm that has been selected for many uses including production of grain, forage, sugar, and biomass for bioenergy. In its native Africa, sorghum grows 4–5 meters tall and many genotypes are photoperiod sensitive, resulting in delayed flowering in long day environments. Upon introduction to temperate locations, photoperiod insensitive varieties that flower early were selected for production of grain^1^. Additionally, shorter grain varieties were selected to reduce lodging and to aid mechanical harvesting. In contrast, sorghum genotypes with longer stems and delayed flowering enhance biomass and sugar production^2, 3^. In sweet sorghum, stem length is associated with higher sugar yield because stems accumulate high levels of sucrose post floral initiation^3–5^. In energy sorghum, 83% of the shoot biomass accumulates in the stem^6^. Therefore, increasing our knowledge of stem growth will aid the improvement of sorghum hybrids for bioenergy production.

Plant height is determined primarily by the length and number of stem internodes. The number of internodes produced by a plant is a consequence of growth duration and the rate of internode production. Quinby and Karper^7^ identified four loci (*Dw1*-*Dw4*) that control internode length by measuring the height of the stem from the ground to the flag leaf. At each *Dw* locus the dominant allele increased internode length. Recessive alleles of *Dw1* and *Dw2* were identified in Milo lines, while recessive alleles of *Dw3* were identified in Kafir backgrounds, and dominant alleles at *Dw4* were only found in broomcorns^7^. *Dw2* was shown to have pleiotropic effects on panicle length, seed weight, and leaf area^8, 9^. In addition to internode length, *Dw3* influences grain yield, tiller number^10^, and leaf angle^11^.

*Dw3* was the first dwarfing gene to be cloned in sorghum^12^. *Dw3* encodes a homolog of the maize *Br2* gene and is an ATP-binding cassette type B1 (ABCB1) auxin efflux transporter. This is in contrast to dwarfing or semi-dwarfing genes in other important crops, such as rice and wheat, which have mutations in genes involved in the gibberellin pathway^13, 14^. *Dw1* was mapped to a region on chromosome 9 between 56.8–57.1 Mb^15^. The gene corresponding to *Dw1* was recently identified as Sobic.009G229800 by map-based cloning^16, 17^. This gene regulates internode cell proliferation^17^ and encodes a putative membrane protein not previously assigned a function^16^. The recessive *dw1* allele in Dwarf Yellow Milo (DYM), first identified by Quinby and Karper^7^, contains a stop codon in exon 2 that results in protein truncation^16^. The *dw1* allele originating from Dwarf Yellow Milo has been used extensively in grain sorghum breeding programs.

*Dw2* has also been used extensively in grain sorghum breeding programs to reduce plant height. *Dw2* is linked to *Ma1*, an important flowering time gene that confers photoperiod sensitivity^1^. *Ma1* is located on chromosome 6 at ~40.3 Mb and encodes PRR37^18^. *Dw2* was previously mapped to a location near *Ma1* at ~42 Mb in several QTL mapping studies^19–22^ and was associated with a SNP marker at 42.7 Mb in a GWAS study^23^. In another study, *Dw2* was suggested to be a histone deacetylase (Sobic.006G067600) based on GWAS analysis^21^. Recessive alleles of *Ma1* and the dwarfing genes were used in the Sorghum Conversion Program to convert tall late flowering landraces from Africa into short, early flowering genotypes that are useful for grain sorghum breeding. The landraces were crossed to BTx406 (*dw1dw2dw3dw4*) to introduce one or more of the recessive alleles at the *Dw* loci into landrace backgrounds^19^. Recent analysis of the sorghum conversion lines has shown that large portions of chromosome 6 have been introgressed from BTx406 into landrace accessions during conversion and that the peak of introgression frequency aligned with *Dw2* ^24^.

In the current study, *Dw2* was map-based cloned using two RIL populations: BTx623 (*dw1Dw2dw3dw4*) x IS3620c (*dw1dw2Dw3dw4*) and BTx642 (*dw1dw2dw3dw4*) x Tx7000 (*dw1Dw2dw3dw4*). *Dw2* was identified as a protein kinase whose closest homolog in Arabidopsis is the kinesin-like calmodulin-binding protein (KCBP)-interacting protein kinase (KIPK), a member of the AGCVIII subfamily that also includes PINOID (PID) and PHOTOTROPIN1 and 2 (PHOT1 and 2).

## Results

### Comparison of DYM and DDYM internode lengths

The recessive *dw2* allele present in Double Dwarf Yellow Milo (DDYM), the original source of *dw2*, arose as a mutation in Dwarf Yellow Milo (DYM)^19, 25^. Comparison of DYM and DDYM stem internode lengths at anthesis showed that the recessive allele of *dw2* in DDYM caused a reduction in the length of every elongated internode compared to the corresponding internodes in DYM (Supplementary Fig. S1). The *dw2* allele found in DDYM was used extensively in U.S. grain sorghum breeding programs and the Sorghum Conversion Program^19^ to reduce the length of stems of sorghum genotypes such as IS3620c and BTx642 that were used in this study to clone *Dw2*.

### QTL Mapping using a RIL population derived from a cross of BTx623 x IS3620c

**Figure 1:**
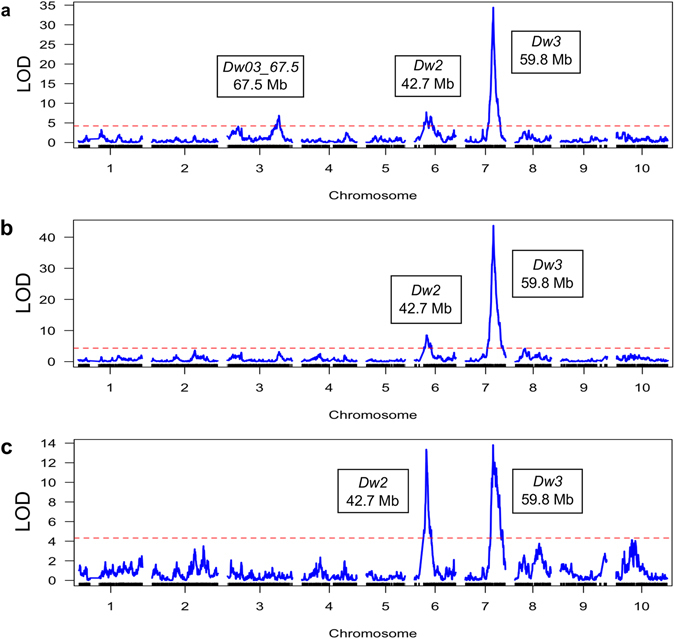
QTL identified using the BTx623 x IS3620c RIL population. The RIL population was grown in the greenhouse and genotyped using DG. Stem length (**a**) was measured from the base of the plant to the base of the panicle. Genetic map generation and QTL mapping were performed in R/qtl using interval mapping (IM). The x-axis is the markers along the chromosomes and the y-axis is the LOD score. The significant QTL peaks are labeled with the *Dw* locus and location (Mb). Stem length (**a**), average internode length (**b**), and the length of the first internode below the peduncle (**c**) are shown.

**Figure 2:**
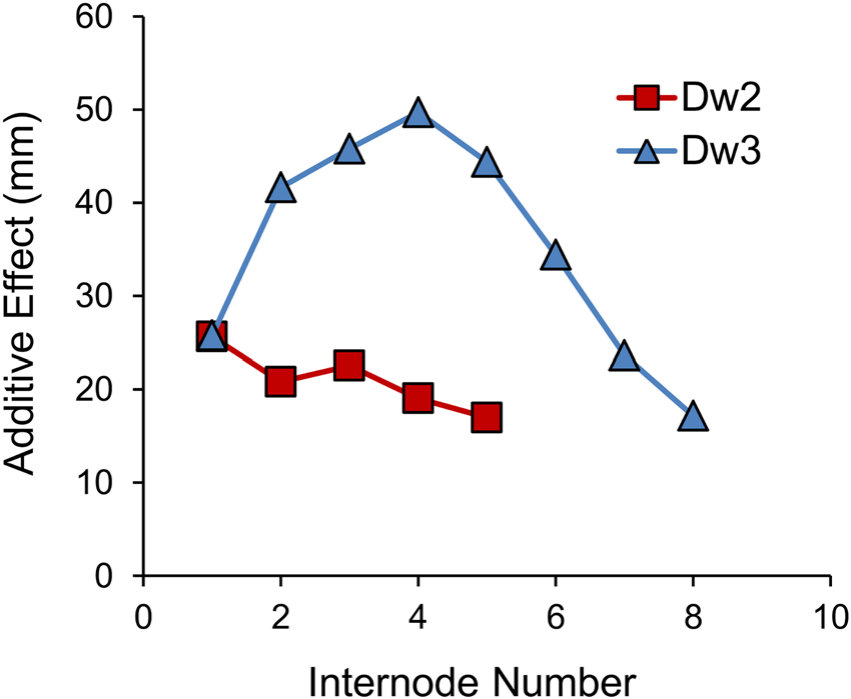
Additive effects of *Dw2* and *Dw3* on the length of each internode (BTx623 x IS3620c RIL population). The RIL population was grown in the greenhouse and the length of each internode (numbered from the peduncle) was measured. Additive effects were determined as part of QTL mapping performed in R/qtl using IM. The BTx623 allele of *Dw2* increases internode length, whereas the IS3620c allele of *Dw3* increases internode length.

QTL for total stem length, average internode length, the length of each internode numbered from the peduncle, and the length of the peduncle were mapped using the BTx623 x IS3620c RIL population (Fig. 1, Supplementary Fig. S2, Table 1). As expected the population segregated for *Dw2* on chromosome 6 (~42.7 Mb) and for *Dw3* on chromosome 7 (~59.8 Mb) and these loci affected both total stem length and internode length. An additional QTL (*Dw03_67*.*5*) at ~67.5 Mb on chromosome 3 affected total stem length (Fig. 1). The influence of *Dw2* and *Dw3* on the length of the eight internodes was analysed to determine if the action of these genes varies with development (Table 1). *Dw3* affected the length of all eight internodes measured. *Dw2* influenced the length of the first five internodes but had minimal impact on the length of internodes 7–8. There is an additional QTL on chromosome 6 (48.6 Mb, *Dw06_48*.*6*) near *Dw2* segregating for the length of the sixth internode below the peduncle. However, the peaks for the fifth and sixth internode are broad and the 2-LOD interval for the peak on chromosome 6 for both internodes includes both *Dw2* and *Dw06_48*.*6* (Table 1, Supplementary Fig. S2). The additive effect of *Dw2* and *Dw3* on internode length varied with internode number (Fig. 2). The additive effect for *Dw2* was highest for the internode immediately below the peduncle. The additive effect of *Dw3* on the length of the same internode was similar to that of *Dw2*. However, *Dw3* influenced the length of internodes formed earlier in development more than *Dw2*. The additive effect of *Dw3* decreased from the sixth to eighth internodes (Fig. 2). QTL for peduncle length did not align with *Dw2* or *Dw3* (Supplementary Fig. S2).

**Table 1:** QTL Segregating for Stem Traits in the BTx623 x IS3620c RIL Population.

Trait	Chr	Peak (bp)	Peak (cM)	LOD	2-LOD Interval		Additive Effect^a^
					Start	Stop	
Total Length	3	67,503,832	136.10	6.89	65,530,485	68,206,300	−12.48
	6	42,691,080	31.14	7.76	42,355,109	46,697,460	−13.29
	7	59,830,285	73.54	34.43	59,654,592	59,867,828	26.00
Average Internode Length	6	42,691,080	31.14	8.52	42,355,109	44,831,591	−20.25
	7	59,830,285	73.54	43.75	59,654,592	59,847,033	41.05
Length Peduncle	2	76,607,596	169.46	4.42	74,943,883	77,320,040	15.37
	3	70,750,399	150.22	13.51	62,718,371	71,404,420	−39.20
	7	59,086,124	68.41	7.73	55,545,487	59,785,398	−31.25
	10	7,100,563	47.03	6.17	5,639,508	48,402,197	−26.97
Length Internode 1	6	42,691,080	31.14	13.35	42,355,109	43,632,616	−25.62
	7	59,785,398	73.44	13.82	59,533,447	60,458,272	25.77
Length Internode 2	6	42,691,080	31.14	6.27	41,934,840	45,943,225	−20.81
	7	59,785,398	73.44	29.72	59,654,592	59,991,087	41.65
Length Internode 3	2	64,347,846	113.94	4.83	63,835,432	64,886,659	9.50
	6	42,691,080	31.14	7.36	42,051,620	45,706,034	−22.53
	7	59,830,285	73.54	36.21	59,631,468	59,847,033	45.81
Length Internode 4	6	42,691,080	31.14	5.71	38,080,498	46,697,460	−19.04
	7	59,830,285	73.54	49.53	59,654,592	59,847,033	49.70
Length Internode 5	3	67,503,832	136.10	4.45	3,482,238	68,957,430	−15.62
	6	42,691,080	31.14	5.60	39,022,638	49,672,003	−16.98
	7	59,830,285	73.54	44.48	59,654,592	59,847,033	44.36
Length Internode 6	3	62,683,672	123.74	5.50	60,818,299	66,423,271	−15.63
	6	48,641,758	50.58	4.83	42,551,078	50,220,562	−13.67
	7	59,830,285	73.54	31.19	59,654,592	59,991,087	34.42
Length Internode 7	1	56,402,777	66.50	6.20	20,256,774	58,060,819	15.54
	7	59,785,398	73.44	16.91	59,481,526	59,991,087	23.64
Length Internode 8	1	56,499,134	66.61	6.10	24,523,367	58,177,975	14.12
	7	59,628,954	72.90	9.82	59,277,216	59,991,087	17.18

Internodes are numbered from the peduncle. Chromosome is abbreviated as “Chr”. ^a^A positive effect indicates that the IS3620c allele increases length.

There was no strong statistical evidence of a genetic interaction between *Dw2* and any of the other loci from the multiple-QTL mapping (MQM) analysis (Supplementary Table S1). For the best model for each phenotype, the only phenotype that included interactions in the model with the highest LOD is the length of internode 7. There are two interactions in this model, one between a QTL on chromosome 5 and *Dw3* (chromosome 7 at 59.8 Mb) and another between a QTL on chromosome 1 and a QTL close to *Dw3* (10.7 cM from *Dw3* at 61.2 Mb) (Supplementary Table S1). The composite multiple-QTL model included both interactions and revealed interesting trends between the internode length traits with internodes further from the peduncle having better support for the two interactions (Supplementary Table S2, Supplementary Fig. S3). Additionally, composite model analysis clarified the effects of the two QTL on chromosome 6. *Dw2* affects the length of internodes 1–6, but starting at internode 4 and continuing through internode 7 the QTL at ~49 Mb on chromosome 6 also affected internode length (Supplementary Table S2, Supplementary Fig. S3).

### *Dw2* fine mapping and gene identification

**Figure 3:**
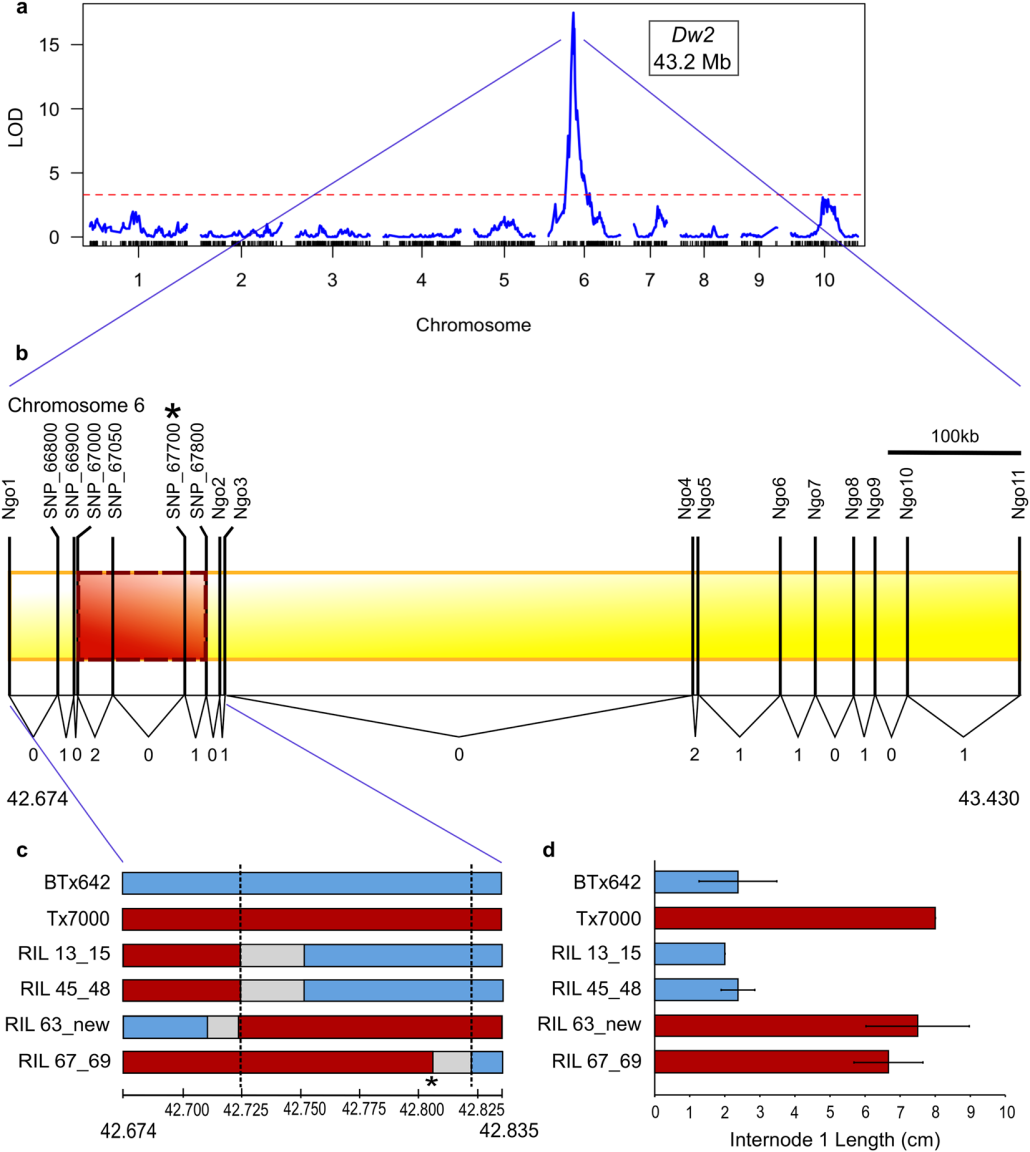
Fine mapping of *Dw2* in the BTx642 x Tx7000 RIL population. (**a**) QTL map of total plant height (2009) with *Dw2* labeled. Plant height was measured as the length of the plant from the base of the stem at ground level to the top of the panicle. Genetic map construction and QTL analysis were performed in R/qtl using IM. The x-axis is the markers along the chromosomes and the y-axis is the LOD value. (**b**) Diagram of fine mapping in BTx642 x Tx7000. The diagram shows the location of the recombination breakpoints in the 2-LOD region in the eight fine mapping lines (numbers at bottom), two of these lines had more than one recombination breakpoint in the region. The markers found through DG using NgoMIV are labeled as “Ngo_”. The markers found with Sanger sequencing are labeled with “SNP_ _” with the last five digits of the gene name. The red, dashed-line box shows the refined region of *Dw2*. For both (**b**) and (**c**), asterisk indicates the approximate location of *Dw2*. (**c**) Diagram of the haplotypes of the four fine mapping lines with breakpoints closest to the refined region. The region between Ngo1 and Ngo3 is shown. Blue indicates that the RIL has the BTx642 allele, red is the Tx7000 allele, and grey is the region where the breakpoint is located. Dashed lines flank the refined region of *Dw2*. (**d**) The length of the first internode below the peduncle in the same lines shown in (**c**). Blue indicates that the line is *dw2* while red is *Dw2*. Average (n = 4) and standard deviation is shown.

*Dw2* was fine mapped in a second RIL population derived from BTx642 x Tx7000 that was expected to segregate for alleles of *Dw2* in a background fixed for recessive *Dw1*, *Dw3*, and *Dw4*. QTL analysis of the BTx642 x Tx7000 RIL population for total plant height revealed a major QTL aligned with *Dw2* as expected (Fig. 3a). The QTL corresponding to *Dw2* showed a peak located on chromosome 6 at ~43.2 Mb. The 2-LOD interval containing *Dw2* in the BTx642 x Tx7000 RIL population spanned a region of ~756 kb on chromosome 6. Eight RILs with recombination breakpoints in this region were identified and targeted for higher resolution analysis of breakpoint locations. Sequence polymorphisms within the target interval identified using high resolution DG analysis and by targeted gene sequencing were used to fine map the breakpoints in the eight fine mapping lines (Fig. 3b). Four RILs with breakpoints closest to *Dw2* were phenotyped in a greenhouse during the winter. Phenotyping in the winter under low light conditions revealed that *Dw2* had a large impact on the length of the internode below the peduncle. As a consequence, RILs containing *Dw2* could be readily distinguished from RILs encoding *dw2* by phenotyping eight plants from each genotype for the length of the internode below the peduncle (Fig. 3c and d, Supplementary Fig. S4). The information from lines with breakpoints delimited the *Dw2* locus to a region spanning ~98.1 kb containing ten genes (Fig. 3, Table 2, Supplementary Table S3). The genes within this region were annotated in Phytozome as encoding a PPR repeat protein, an rRNA N-glycosylase, an F-box protein, a glycogen branching enzyme, a phosphatase, a histone deacetylase, a kinase, and three genes of unknown function.

**Table 2:** Genes in the Delimited Region of *Dw2*.

Gene	Description	Location (v3.1)
Sobic.006G067000	PPR repeat	42,723,881–42,725,688
Sobic.006G067050	Unknown	42,751,421–42,752,998
Sobic.006G067100	rRNA N-glycosylase	42,753,303–42,756,717
Sobic.006G067150	Unknown	42,758,806–42,759,413
Sobic.006G067200	Unknown	42,760,512–42,761,535
Sobic.006G067300	F-box domain	42,769,007–42,770,832
Sobic.006G067400	1,4-alpha-glucan branching enzyme; Calcineurin-like phosphoesterase	42,774,078–42,778,987
Sobic.006G067500	Calcineurin-like phosphoesterase; Ser/Thr protein phosphatase family protein; Prespore protein DP87	42,781,244–42,785,442
Sobic.006G067600	Histone deacetylase	42,785,485–42,802,516
Sobic.006G067700	Ribosomal protein S6 kinase; Protein tyrosine kinase	42,803,037–42,807,134

### Sequence analysis of genes in the *Dw2* locus

The gene corresponding to *dw2* is expected to contain a mutation(s) that decreases function; therefore, all of the genes in the delimited *Dw2* locus (Table 2) were sequenced from DYM and DDYM. Only one polymorphism was found in the delimited *Dw2* locus that distinguished DYM from DDYM, an indel in Sobic.006G067700 located in the first exon at 549 bp that causes a frameshift resulting in a stop codon at 573 bp. This mutation changed the amino acid sequence after E183 resulting in a truncated polypeptide containing 190 amino acids instead of the 809 amino acids present in the full-length protein. The indel mutation in Sobic.006G067700 that causes protein truncation was also present in BTx642 and IS3620c, genotypes that acquired *dw2* by introgression from DDYM, and not present in BTx623 (*Dw2*) and Tx7000 (*Dw2*) (Tables 3 and 4). None of the parental lines contain polymorphisms in the coding region of the histone deacetylase (Sobic.006G067600), a gene previously proposed as a candidate for *Dw2* ^21^. A number of sequence variants in the *Dw2* delimited region were identified that distinguished the parental mapping lines (Supplementary Table S4); however, none of these variants differentiated DYM (*Dw2*) from DDYM (*dw2*), the source of the recessive allele of *dw2*.

### *Dw2* alleles in sorghum germplasm

The distribution of *Dw2* alleles in historically important sorghum genotypes was investigated by sequencing Sobic.006G067700 from the genotypes listed in Tables 3 and 4. Many of the genotypes identified by Quinby and Karper^7^ as *dw2* contain the indel in Sobic.006G067700 derived from DDYM. For example, 80 M is a maturity standard with the reported genotype *dw1dw2Dw3dw4* ^1^. 80 M and the other maturity standards were selected from a cross of Early White Milo (*Dw2*) and DDYM (*dw2*) and contain the DDYM *dw2* allele (Table 4). BTx406 also contains the DDYM *dw2* allele, consistent with a previous study showing that the *dw2* allele in BTx406 was derived from DDYM based on pedigree analysis and haplotype-graphical genotyping^19^. BTx406 was used in the conversion of IS3620 to the early flowering short genotype IS3620c, explaining why IS3620c contains the DDYM *dw2* allele. SC170 and BTx642 (formerly B35) were also crossed to genotypes containing the DDYM *dw2* allele during their construction^26^. Several genotypes contained polymorphisms in exons that changed the protein sequence encoded by Sobic.006G067700. However, SIFT^27^ analysis predicted that those polymorphisms would be tolerated and not disrupt function (Table 3). As expected, the haplotypes of the two *dw2* recessive RIL population parents, BTx642 and IS3620c, were the same as the progenitor lines DDYM and BTx406. The haplotypes of the two *Dw2* dominant RIL population parents, BTx623 and Tx7000 were the same as the progenitor line Texas Blackhull Kafir (Table 4). Six other genotypes identified by Quinby and Karper^7^ as *Dw2* also encoded full length proteins (Table 4). Hegari was reported to contain a recessive *dw2* allele^1, 7^. However, subsequent analysis showed that Hegari has the dwarfing genotype *Dw1*, *Dw2*, *dw07_55*.*1*, *Dw3* ^16^. Consistent with the revised genotype, Hegari encoded a full length Dw2 protein and lacked the DDYM indel in Sobic.006G067700 that disrupts protein function (Table 4).

**Table 3:** Polymorphisms in Sobic.006G067700.

Number	Polymorphism	Location	Region	Result	SIFT
1	SNP; C > T	−138 bp	5′UTR		
2	11 bp INDEL	−132– > –122bp	5′UTR		
3	INDEL; GA > –	549 bp	Exon 1	Stop codon at 573 bp	
4	SNP; G > A	650 bp	Exon 1	Glycine > Aspartic Acid	0.09 = tolerated
5	SNP; C > T	875 bp	Exon 1	Proline > Leucine	0.10 = tolerated
6	SNP; A > C	1279 bp	Exon 1	Isoleucine > Leucine	0.17 = tolerated
7	SNP; G > A	2561 bp	Exon 2	Cysteine > Tyrosine	0.11 = tolerated

**Table 4:** Selected Genotypes Scored at the Polymorphisms Listed in Table 3.

Line	Dw2	1	2^a^	3	4	5	6	7
BTx623	*Dw2*	C	+	GA	G	C	A	G
IS3620c	*dw2*	T	−	−	A	C	C	A
Tx7000	*Dw2*	C	+	GA	G	C	A	G
BTx642	*dw2*	T	−	−	A	C	C	A
Standard Yellow Milo	*Dw2*	T	−	GA	A	C	C	A
Dwarf Yellow Milo	*Dw2*	T	−	GA	A	C	C	A
Double Dwarf Yellow Milo	*dw2*	T	−	−	A	C	C	A
80 M	*dw2*	T	−	−	A	C	C	A
SC170	*dw2*	T	−	−	A	C	C	A
BTx406	*dw2*	T	−	−	A	C	C	A
Texas Blackhull Kafir	*Dw2*	C	+	GA	G	C	A	G
Hegari	*dw2* ^b^	T	−	GA	G	T	C	A
Early White Milo	*Dw2*	C	+	GA	G	C	A	G
Spur Feterita	*Dw2*	C	+	GA	G	C	C	A
Sumac	*Dw2*	C	+	GA	G	C	A	G

^a^The plus sign indicates that the genotype lacks the deletion, while the minus sign indicates that the genotype has the deletion. ^b^Hegari is listed as recessive *dw2* by Quinby and Karper^7^; however, a QTL mapping F_2_ population of Hegari and 80 M segregated for *Dw2*, with the increase in length coming from Hegari^16^.

### Dw2 is homologous to the AGCVIII protein kinase KIPK

**Figure 4:**
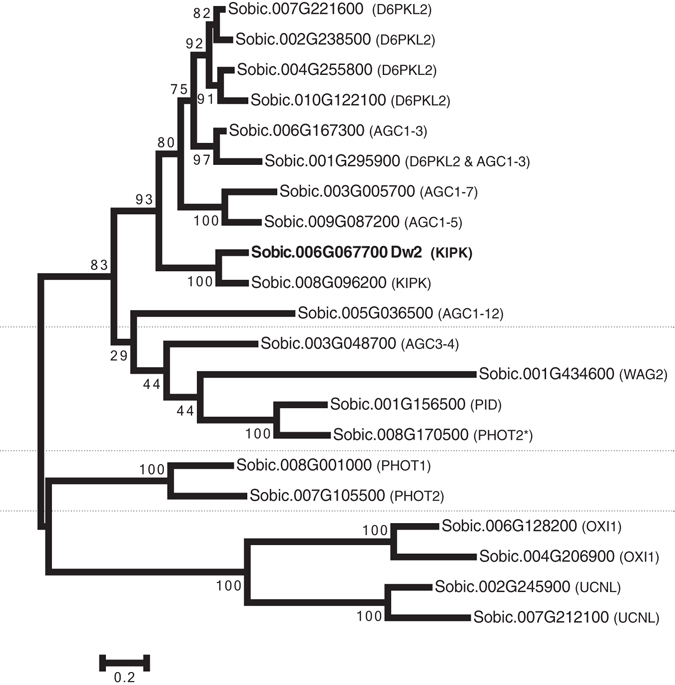
Phylogenetic tree of the AGCVIII subfamily in sorghum. The tree of the 21 sorghum AGCVIII genes was generated in MEGA6 using Maximum Likelihood. Dw2 is bolded. The four different groups, AGC1-4, are labeled and colored. The names in parenthesis are the best hit from a BLAST search of the Arabidopsis genome using that sorghum gene as a query. *The best hit for Sobic.008G170500 is PHOT2 but the score is much lower than Sobic.007G105500 to PHOT2 (203.4 vs. 1122.1 for the Dual Affine Smith Waterman alignment score). Further, Sobic.008G170500 is the best BLAST match of the maize PID homolog, BARREN INFLORESCENCE2, in sorghum.

Fine mapping, sequence analysis, and gene annotation indicates that Dw2 is a protein kinase encoded by Sobic.006G067700 (Phytozome). Genes in other plants with the greatest sequence similarity to Sobic.006G067700 include LOC_Os12g29580 (rice), GRMZM2G412524 (maize), GRMZM2G128319 (maize), and At3G52890 (Arabidopsis) (Phytozome). At3G52890 encodes an ACGVIII kinase called KIPK, a KCBP-interacting protein kinase^28^. In Arabidopsis there are 23 members of the AGCVIII kinase subfamily that has been further subdivided into four groups, AGC1–AGC4. KIPK and D6 PROTEIN KINASE/D6 PROTEIN KINASE LIKEs (D6PK/D6PKLs) are members of the AGC1 group. A BLAST search of the Arabidopsis AGCVIII kinase gene family to the sorghum genome identified 21 sorghum homologs (Supplementary Table S5). Among these genes, Dw2 was the best BLAST hit for the Arabidopsis KIPK1, KIPK2 (AGC1–9) and AGC1-8. KIPK1 and KIPK2 also aligned well with a related gene in sorghum, Sobic.008G096200. Since the correspondence between AtKIPK1, AtKIPK2 and the two sorghum homologs could not be assigned, we designated Dw2 as SbKIPK and Sobic.008G096200 as SbKIPK-like. The relationship among the 21 members of the sorghum AGCVIII subfamily was analysed by constructing a phylogenetic tree (Fig. 4). The sorghum genes clustered into four groups, as in Arabidopsis, though the closest sorghum homolog to AGC1-12 (Sobic.005G036500) groups with the AGC3s. If this gene is excluded from the sorghum AGC1 subfamily, then sorghum has three fewer members of the AGC1 group than Arabidopsis. Interestingly, while similar phylogenetic trees of the Arabidopsis AGC1 subfamily showed KIPK1 and KIPK2 grouping with AGC1-8^29–31^, the sorghum AGC1 family has only two genes on that branch, Sobic.006G067700 (Dw2, SbKIPK) and Sobic.008G096200 (SbKIPK-like) (Fig. 4). The sorghum AGC1 group also includes a cluster of four sorghum genes that correspond to the four Arabidopsis genes that encode D6PK/D6PKLs. The sorghum AGC3 group has five members, including the AGC1-12 homolog, with two genes matching with PID and one gene corresponding with the WAGs. The remaining two groups of sorghum genes corresponding to AGC2 and AGC4 are similar to Arabidopsis (Fig. 4 & Supplementary Table S5).

Plant AGC kinases contain a catalytic core consisting of 12 conserved subdomains^32^. A comparison of Dw2 (Sobic.006G067700) with KIPK and other members of the AGC1-kinase group showed that Dw2 contains a conserved GxGxxG sequence in the P-loop of sub-domain I of the N-lobe, an activation segment in the C-lobe that includes the Mg++ binding sequence DFDLS, an insertion domain typical of plant AGC-kinases, and a T-loop and activation domain [SxxSFVGTxYxAPE] that is a site of phosphorylation^32^ (labelled in Supplementary Fig. S5). The protein has a C-terminal FxxF sequence found in many AGC-kinases that binds 3-phosphoinositide-dependent kinase 1 (PDK1), a highly conserved member of the AGC kinase family that phosphorylates several AGC kinases^30^. AGC kinases vary significantly in the length, sequence, and function of their N-terminal domains that often mediate interaction with other proteins. KIPK1 and 2 and AGC1-8 have N-terminal domains of 546–549 amino acids, significantly larger than other members of the AGC1 kinase subfamily^32^. When the N-terminal 423 amino acid domain of Dw2 was used to search for matches in the Arabidopsis genome (Phytozome), it aligned best with the N-terminal domain of KIPK and next best to KIPK2 (AGC1-9). Multiple sequence alignment of Dw2, rice and maize homologs of Dw2, and Arabidopsis KIPK showed regions of sequence similarity throughout the N-terminal domain and several deletions relative to Arabidopsis KIPK that explain the difference in overall length of the N-terminal protein sequences (423 versus 545 amino acids) (Supplementary Fig. S5).

### Expression of *Dw2*

**Figure 5:**
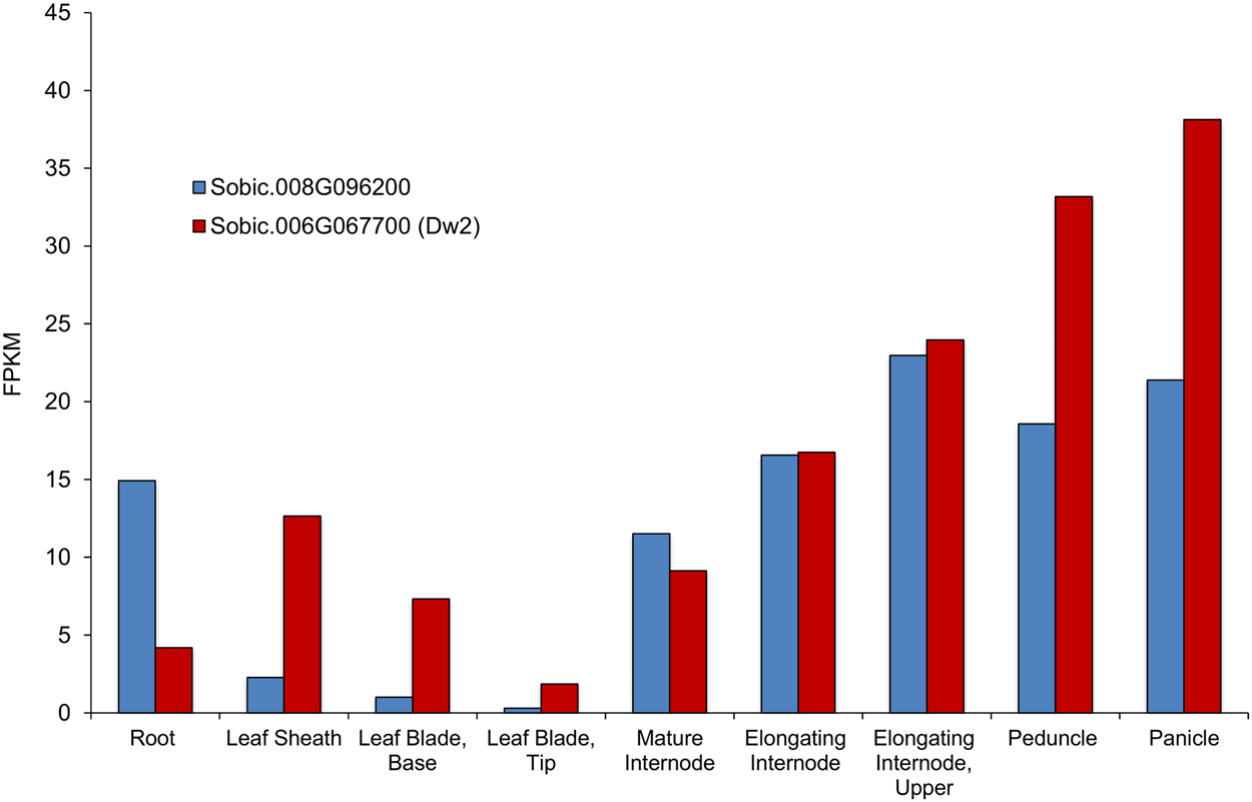
Expression of *Dw2* and Sobic.008G096200 in various tissues. Gene expression data is from the publicly available RNA-seq GeneAtlas on Phytozome v11. Tissues are from BTx623 (dominant *Dw2*) at 44 Days after Emergence (DAE). The leaf tissue was taken from the last ligulated leaf, so the base is still growing whereas the tip is maturing.

*Dw2* RNA abundance was examined in tissues of BTx623 (*Dw2*) by analysis of RNAseq profiles that are part of the sorghum RNA Atlas (Phytozome). *Dw2* is annotated as having two transcripts that differ in the 5′UTR. The primary transcript (Sobic.006G067700.2) has a UTR with no introns that extends 537 bp before the start codon, while the secondary transcript (Sobic.006G067700.1) has one intron and extends 923 bp. The analysis of *Dw2* expression shown in Fig. 5 utilized tissues collected from plants at ~10 days post-floral initiation, when upper leaves, leaf sheaths, internodes, nascent panicles and peduncles are growing. The expression of *Dw2* was relatively high in developing panicles, peduncles, growing internodes and leaf sheaths, with lower expression in fully expanded internodes, leaf blades and the lower portion of the root system that includes root tips and fully elongated roots (Fig. 5 & Supplementary Fig. S6). The expression of sorghum KIPK-like (Sobic.008G096200) was higher than *Dw2* in roots and lower in leaf tissues, the peduncle, and panicle (Fig. 5).

## Discussion

In this study, *Dw2*, an important dwarfing locus used in grain sorghum breeding, was mapped as a QTL in two populations. Using map-based cloning, the gene corresponding to *Dw2* was identified as a protein kinase whose closest homolog in Arabidopsis is KIPK, a member of the AGCVIII protein kinase family.

*Dw2* QTL analysis and fine mapping were performed using two different RIL populations. In the first population derived from BTx623 x IS3620c, alleles of the dwarfing loci *Dw2* and *Dw3* were segregating. Analysis of average internode length identified a QTL aligned with *Dw2* at ~42.7 Mb on chromosome 6 and a QTL corresponding to *Dw3* located on chromosome 7 at ~59.8 Mb. *Dw2* was the only dwarfing (*Dw*) locus segregating in the second population derived from BTx642 x Tx7000, genotypes recessive for *dw1dw3dw4*. Indeed, the only QTL segregating for total height in this population was a QTL corresponding to *Dw2* (~43.2 Mb). The location of the *Dw2* QTL mapped in this study corresponds to most previous reports of the location of *Dw2* ^19, 21^. Higgins *et al*.^22^ also identified QTL for plant height in this region of chromosome 6 with peaks at 44.3–44.5 Mb or 42.1 Mb depending on the population and QTL model. The authors suggested that variation in QTL location was due to the linkage between *Dw2* and *Ma1* since both influence plant height^22^. In the current study, the influence of *Ma1* alleles is minimal because the BTx642 x Tx7000 RIL population is segregating for a weak allele and null allele of *Ma1*, respectively^33^, and BTx623 and IS3620c each contain null alleles of *Ma1* ^18, 19^. During the analysis of *Dw2* a nearby QTL located at 48.6 Mb on chromosome 6 was identified that modified the length of internode 6 according to single QTL mapping. MQM revealed that this QTL also affected the length of internodes 4–7; however, *Dw2* had a greater impact on the length of the fourth and fifth internode. This additional QTL could also have confounded the location of *Dw2* in the study of Higgins *et al*.^22^.

QTL analysis in the BTx623 x IS3620c population showed that *Dw2* and *Dw3* influence internode length differentially during development. *Dw2* had the greatest additive effect on the length of the internode immediately below the peduncle. The additive effects of *Dw2* and *Dw3* on the length of this internode were similar. The influence of *Dw2* gradually decreased in the internodes below the top internode and there was no detectable impact of *Dw2* on the length of internodes 7–8 below the peduncle in this population. *Dw3* had a much greater effect than *Dw2* on internodes 2–5 below the peduncle with reduced but significant impact on the length of internodes 6–8 (Table 1, Fig. 2). Similarly, in maize, *Br2*, the homolog of *Dw3* that encodes an ABCB1 auxin transporter, had a greater influence on elongation of the lower stem internodes compared to upper internodes that elongate post-floral initiation^12^. RILs from the BTx642 x Tx7000 population that are null for *Dw3* and differ in *Dw2* alleles showed a large difference in length of the internode below the peduncle when grown in low light in the greenhouse during the winter (Supplementary Fig. S4). A comparison of the yellow milos (DYM: *dw1* ***Dw2*** *Dw3dw4* and DDYM: *dw1* ***dw2*** *Dw3dw4*) showed that *Dw2* has an effect on the length of all of the ~12 internodes produced by plants grown in the greenhouse during the fall under short day conditions (Supplementary Fig. S1). Taken together these results indicate that *Dw2* affects the length of internodes produced by plants during the vegetative phase and the last 6–7 internodes produced after floral initiation. The relative impact of *Dw2* on the length of sorghum stem internodes depends on genetic background, stage of development, and environmental conditions.

Fine mapping narrowed the region encoding *Dw2* to a ~98.1 kb region of chromosome 6 containing ten genes. Fine mapping was more effective in the BTx642 x Tx7000 RIL population compared to the BTx623 x IS3620c RIL population even though the population size was smaller. Map-based cloning of *Ma1* (*PRR37*), a gene located near *Dw2* on chromosome 6 (40.3 Mb), was also more effective in one of the three populations used in that study^18^. The reason for differences in recombination efficiency among populations used to map *Ma1* and *Dw2* is unknown. However, it is possible that sequence divergence or differences in chromatin/DNA methylation in this region of chromosome 6 between parental genotypes affects local recombination frequency. In both studies, the most useful population for fine mapping involved parental lines of the durra race that were crossed to parental lines of the kafir race (BTx642 (durra) xTx7000 (kafir) (*Dw2*); 100 M (durra) x Blackhull kafir (*Ma1*)). Larger populations and additional crosses will be required to determine if the association between genetic background and local recombination frequency is significant.

One of the ten genes in the delimited *Dw2* locus encoded a histone deacetylase that was previously suggested to be a candidate for *Dw2* ^21^. However, the deacetylase did not contain polymorphisms in the coding regions that distinguish the parental genotypes used for fine mapping, or DYM (*Dw2*) and DDYM (*dw2*). DDYM was reported to have originated as a shorter mutant in a field of DYM^25^. Thus, these two yellow milos should be isogenic except at *Dw2*. All of the other genes in the delimited *Dw2* region were sequenced from DYM and DDYM. Only the kinase encoded by Sobic.006G067700 had a polymorphism that distinguished DDYM from DYM in the delimited *Dw2* locus. This polymorphism resulted in a frameshift mutation and a premature stop codon in the first exon. This results in a protein of only 190 amino acids instead of 809 amino acids found in DYM. The kinase domain is located between 424–763 amino acids; therefore, the mutant protein found in DDYM would lack kinase activity.

The closest homolog of sorghum Dw2 in Arabidopsis is KIPK, a member of the AGC family of kinases. The AGC family is named after the cAMP dependent protein kinases, cGMP dependent protein kinases, and protein kinase C and also includes PDK1 and the ribosomal protein S6 kinases. The plant-specific AGCVIII subfamily includes PID, PHOT1 and 2, and the D6PK/D6PKLs^34^. Each of these kinases has been shown to regulate auxin efflux transporters, including ABCB1 and PIN1, with PHOT1 and 2 doing so in a blue-light dependent manner^32, 35^. In Arabidopsis, KIPK has a close homolog, KIPK2 (also known as AGC1-9 and At2g36350) and the closely related kinase, AGC1-8^30, 36^. In sorghum, Dw2 has one closely related homolog, Sobic.008G096200, and these two genes form their own branch on the phylogenetic tree (Fig. 4). As some of the members of the AGCVIII subfamily have been shown to regulate auxin transport, Dw3, the sorghum homolog of Arabidopsis ABCB1, was initially considered a potential target of Dw2 action. However, while *Dw2* was expressed in growing internodes, MQM analysis provides no genetic evidence for interaction between *Dw2* and *Dw3*. Furthermore, the *Dw2* allele positively affects the length of the upper most internode in a *dw3* background, indicating that Dw2 can act at least partially through pathways independent of Dw3.

In Arabidopsis KIPK was so named due to its interaction with KCBP, a plant-specific kinesin-like calmodulin binding protein that functions in cell division and trichome formation^28^. KCBP has a C-terminal motor and calmodulin-binding domain, and is unusual among kinesins in its ability to interact with microtubules and with actin, the latter interaction mediated by a MyTH4-FERM tandem that occurs in myosin^37^. Type-VI kinesin-14 dimers in *Physcomitrella patens*, homologs of KCBP, are highly processive, and transport vesicles/cargo long distances when clustered^38^. KCBP contains a calmodulin binding domain and is down-regulated by calcium via calmodulin as well as the KCBP interacting Ca^2+^-binding protein (KIC)^39, 40^. While KIPK did not phosphorylate the N-terminal end of KCBP under experimental conditions, it is possible that it phosphorylates KCBP under other conditions, and it is possible that KCBP transports KIPK within the cell^28^.

Subsequent work has also shown that Arabidopsis KIPK1 and 2 directly interact with members of the proline-rich extensin-like receptor-like kinase (PERK) family, specifically PERK8, 9, 10, and 13^36^. Other PERK-genes, such as PERK1, mediate growth inhibition, possibly in response to cell wall signals^41^. In Arabidopsis, KIPK1 and 2 double mutants did not produce shoot phenotypes although there were differences in root elongation when plants were grown on elevated sucrose^36^. Different parts of the N-terminal domain of KIPK1 and 2 mediate the direct interactions with KCBP and the various PERKs^36^. The 423 amino acid N-terminal sequence of Dw2 aligned well with the ~545 amino acid N-terminus of KIPK despite several deletions that account for the difference in overall length of this domain. The sequence similarity of the N-terminal domains of KIPK and Dw2 indicates that Dw2 has likely retained the ability to interact with one or more members of the PERK family. The best BLAST hits to Arabidopsis PERK8 and 10 (At5g38560 and At1g26150, respectively) in sorghum (Sobic.003G100700, Sobic.003G289800, and Sobic.009G000300) were expressed in stem internodes (Phytozome). Therefore, it will be of interest to determine if Dw2 interacts with sorghum PERK8 or 10 homologs.

If Dw2, like Arabidopsis KIPK, interacts with PERKs and KCBP, the interactions with these proteins may modulate growth regulation and serve other regulatory functions. For example, because KCBP transports vesicles/cargo long distances^38^ potential Dw2 interactions with PERKs and KCBP in sorghum could regulate growth and the flow of materials to the cell wall during and after organ elongation. Alternatively, in trichomes KCBP has been found to organize cytoskeleton components^37^, thus KIPK may be involved cytoskeletal regulation that is associated with cell elongation. This more general coordinating function may explain why Dw2 is expressed in growing zones of leaf blades, leaf sheaths, stems, and panicles. Lack of growth phenotypes in all organs where *Dw2* is expressed (i.e., peduncle) could be due to the presence of a second KIPK-like gene in sorghum (Sobic.008G096200). In fact, Sobic.008G096200 is more highly expressed than *Dw2* in the roots, and both genes are highly expressed in the panicle, peduncle, and internodes (Fig. 5). One other possibility could be that KIPK is involved in a PERK signalling pathway. Another member of the PERK family, PERK4, has been shown to regulate cell elongation in roots as part of an abscisic acid (ABA) signaling pathway^42^.

While Dw2 is a homolog of Arabidopsis KIPK, Dw2 has an important role in regulating stem length in sorghum, a function not observed in Arabidopsis KIPK mutants^36^. This may be because grass stem growth occurs by sequentially elongating internodes adjacent to intercalary meristems located just above nodes, a mode of stem growth that is unique to grasses. The first sorghum dwarfing locus cloned, *Dw3*, also had a more severe stem phenotype than mutants affecting the Arabidopsis homolog, ABCB1. Multani *et al*.^12^ showed that mutation of *Dw3*, an auxin efflux carrier, results in short internodes in sorghum whereas the corresponding ABCB1 single mutant in Arabidopsis had little effect on stem length^43^. Knoller *et al*.^44^ showed that brachytic2, the maize homolog of sorghum Dw3, is expressed in stem nodes but not in stem internodes, whereas Arabidopsis lacks intercalary meristems. This difference in physiology between Arabidopsis and the grasses helps explain the differences in ABCB1 mutant phenotypes. It may also explain the differences in phenotypes between the Dw2 and KIPK mutants in sorghum and Arabidopsis, respectively. Alternatively, the difference in phenotype could be due to differences in functional redundancy and/or expression within the AGCVIII subfamily.

*Dw2* has been used extensively in grain sorghum breeding in the U.S. to create lines and hybrids with reduced stem length. A recessive allele of *dw2* derived from DDYM was used in the Sorghum Conversion Program to reduce the height of lines that were being converted for use in temperate grain sorghum breeding programs^19^. *Dw2* is linked to *Ma1* (*PRR37*), another important gene in grain sorghum and energy sorghum development^18^. In addition to its historical significance, a better understanding of *Dw2* function may enable the design of improved sorghum crops.

## Methods

### Phenotypic Analysis of DYM and DDYM Stems

The progenitor genotypes Dwarf Yellow Milo (DYM; *Dw2*) and Double Dwarf Yellow Milo (DDYM; *dw2*) were grown to examine the internode length phenotypes caused by the two *Dw2* alleles. For each genotype, three plants were individually grown in 3.8-gallon pots (Custom2000) containing Metro Mix 900 (Sun Gro Horticulture) with supplemental fertilizer (Peters 20-20-20) in the greenhouse in short days during the fall. At anthesis, the plants were harvested and the total stem length and length of each internode were measured.

### QTL Mapping of *Dw2* in a RIL population derived from BTx623 and IS3620c

The BTx623 x IS3620c RIL population was used for mapping *Dw2* ^45^. Seed for the population was obtained from the USDA-ARS Plant Genetic Resources Conservation Unit (Griffin, GA). BTx623 is *dw1Dw2dw3dw4* and IS3620c is *dw1dw2Dw3dw4*; therefore, the population segregated for both *Dw2* and *Dw3*. The population (n = 380) was grown in the greenhouse in the summer of 2013 with natural day lengths. Three plants of each RIL were grown per pot, one pot per line in the same manner as DYM and DDYM. Plants were harvested at grain maturity. For each plant, the total length of the plant (base of the plant to the base of the panicle) and the length of each internode and peduncle were measured. Internodes were numbered from the peduncle. Plants differed for flowering time, with earlier flowering lines producing fewer elongated internodes. As a consequence, the 6^th^, 7^th^, and 8^th^ internodes below the peduncle had smaller sample sizes than the other traits measured (n = 375, n = 356 and n = 296, respectively). Genotyping and genetic map construction (n = 398) were performed as described in Truong *et al*.^46^ except the DG marker sequences were mapped to version 3 of the sorghum reference genome assembly (*Sorghum bicolor* v3.1 DOE-JGI, http://phytozome.jgi.doe.gov/), using BWA^47^, and INDEL realignment and joint variant calling were performed with the GATK using the naive pipeline of the RIG workflow^48–51^. QTL mapping was performed in R/qtl using interval mapping (IM) with 1000 permutations and an α = 0.05^52^. Both the genetic map and QTL mapping were performed as an F_7_ instead of a RIL population due to excess heterozygosity.

MQM was performed using the same phenotypes, except peduncle length, and genotypes that were used for IM, except the genetic map was thinned to obtain a marker set with at least 1 cM spacing between markers. Also, measurements of the length of each internode, average internode length, and total internode length were normalized using Empirical Quantile Normal Transformation prior to QTL mapping with R/qtl^52–54^. Penalties (main effect, heavy interaction, and light interaction) for all normalized phenotypes were calculated from 25,000 permutations of two-dimensional genome scans using the TIGGS-HPC cluster at Texas A&M; penalties calculated were negligibly different between phenotypes (i.e. same to the tenths place). Significant QTL identified from an initial IM analysis (alpha = 0.05, main effect LOD = 3.2) were used to seed multiple-QTL model selection analysis (maximum number of QTL in a model was restricted to 7; main effect LOD = 3.2, heavy interaction LOD = 4.3, light interaction LOD = 1.9)^52, 54^. The best scoring multiple-QTL model from model selection of each phenotype was then merged into a composite multiple-QTL model. The composite multiple-QTL model was generated by merging all overlapping 2-LOD intervals into one QTL and designating the position of the MLOD (maximum LOD) marker as the QTL position^55^, where loci with an epistatic interaction were merged independently of strictly additive loci.

### QTL Mapping of *Dw2* in a RIL population derived from BTx642 and Tx7000

BTx642 is *dw1dw2dw3dw4* and Tx7000 is *dw1Dw2dw3dw4*; therefore, the population derived from a cross of these genotypes will segregate for alleles of *Dw2*. The BTx642 x Tx7000 RIL population (n = 89) was grown in the field in the spring and summer of 2009. It was planted in a Norwood silty clay loam (fine-silty, mixed (calcareous), thermic Typic Udifluvent) in duplicate in a randomized block design at the Texas A&M Research Farm located near Snook, TX on 03/04/2009. The blocks were arrayed in 20 rows 4.6 m long and spaced 76 cm apart with two buffer rows on each end of the block. Each block was offset from the next by approximately 1.5 m. The plants emerged on 08/04/2009 and were thinned to a within-row spacing of 10 cm at 16 days after emergence (DAE). The average daily maximum temperature was 33.3 °C and the average daily minimum temperature was 21.1 °C. The population received 24.9 cm of natural rainfall during the growing season with supplemental flood irrigation as needed. The population was harvested on 23/06/2009 (76 DAE), approximately at anthesis for the population. Three plants of each RIL and parental lines from each of two replicates were harvested. For QTL mapping, the average of the two replications was used. Plants were phenotyped for total height, which was measured from the base of the plant to the top of the panicle.

DNA was extracted from leaf tissue harvested from each RIL and processed using ZR Plant/Seed DNA MiniPrep (Zymo Research). Digital Genotyping (DG) was performed as previously described^20^ using the enzyme NgoMIV to digest genomic DNA. Reads were mapped to the reference genome and variants were processed as described for the BTx623 x IS3620c RIL population. The genetic map was constructed using R/qtl (n = 93) after removing any markers that did not define a recombination breakpoint. QTL mapping was also performed in R/qtl using IM with 1000 permutations and an α = 0.05^52^.

### Fine Mapping of *Dw2*

The BTx642 x Tx7000 RIL population was used for fine mapping *Dw2*. Lines that had recombination breakpoints in or near *Dw2* were used to delimit the locus to the extent possible using additional DG genotypes and SNPs identified by Sanger sequencing genes in the region. Primers used for Sanger sequencing are listed in Supplementary Table S6. All PCR amplification was done with Phusion^©^ High-Fidelity DNA Polymerase (New England BioLabs, Inc.) using the standard conditions. The PCR product was gel purified using QIAquick Gel Extraction Kit (Qiagen) and prepared for capillary sequencing with BigDye^©^ Terminator v3.1 Cycle Sequencing Kit (Applied Biosystems) using standard reaction conditions. Sequencing was performed with the ABI 3130xl Genetic Analyzer (Applied Biosystems) and the results were analyzed with Sequencher v4.8 (Gene Codes Corp.).

RILs with recombination breakpoints in the delimited *Dw2* region were grown to confirm stem and internode length phenotypes. Two pots containing two plants from each RIL were grown in two different greenhouses for a total of eight plants per RIL; otherwise the RILs were grown in the same manner as DYM and DDYM. At anthesis, the plants were harvested and the total length of the stem (measured from the base of the plant to the base of the panicle) and the length of each internode were recorded.

### Sequencing of Genes in the Genomic Region Spanning *Dw2*

Once the region encoding *Dw2* was delimited to the extent possible with available genetic resources, the genes in this region were sequenced to search for functional mutations that distinguish DYM (*Dw2*) from DDYM (*dw2*). The genes in the *Dw2* locus were identified using the sorghum reference genome version 3.1 gene set (*Sorghum bicolor* v3.1 DOE-JGI, http://phytozome.jgi.doe.gov/). The primers for sequencing the genes are listed in Supplementary Table S7 and capillary sequencing was performed as with fine mapping SNPs. DDYM was identified as a short plant in a field of DYM and alleles of *Dw2* differentiate the two genotypes^25^. For Sobic.006G067600 only the exons were sequenced, for all other genes, the entire gene was sequenced. Sobic.006G067700 was further sequenced in the other important breeding lines to examine the distribution and extent of allelic variation in *Dw2*.

### Whole Genome Sequencing

Whole genome sequencing was used to identify polymorphisms that distinguish the parents of the two populations used to map *Dw2*. Tx7000 and BTx642 seeds were obtained from Dr. W.L. Rooney (Dept of Soil and Crop Sciences, TAMU). IS3620c seed (PI 659986 MAP) was obtained from the USDA-ARS Plant Genetic Resources Conservation Unit (Griffin, GA). Seeds were soaked in 20% bleach for 20 minutes and washed extensively in distilled water for one hour. Seeds were germinated on water-saturated germination paper in a growth chamber (14 hr light; 30 °C/10 hr dark; 24 °C). Genomic DNA was isolated from 8-day old root tissue using a FastPrep DNA Extraction kit and FastPrep24 Instrument (MP Biomedicals LLC, Solon, OH, USA), according to the manufacturer’s specifications. DNA template (350 bp average insert size) was prepared using a TruSeq® DNA PCR-Free LT Kit, according to the manufacturer’s directions. Paired-end sequencing (125 × 125 bases) was performed on an Illumina HiSeq2500. Sequence reads were mapped to version 3 of the sorghum reference genome assembly (*Sorghum bicolor* v3.1 DOE-JGI, http://phytozome.jgi.doe.gov/), using BWA v0.7.12^47^. Base quality score recalibration, INDEL realignment, duplicate removal, joint variant calling, and variant quality score recalibration were performed using GATK v3.3 with the RIG workflow^48–51^. Whole genome sequence of Tx7000, BTx6424, and IS3620c are available at the Sequence Read Archive (www.ncbi.nlm.nih.gov/sra).

### Protein Sequence Analysis

Each of the AGCVIII proteins in Arabidopsis was aligned with the sorghum genome using BLAST and the best hits were recorded. The resulting sorghum AGCVIII protein family was used to make a phylogenetic tree in MEGA6^56^. The sequences were aligned using the MUSCLE algorithm^57, 58^. The tree was estimated using maximum likelihood with the substitution model developed by Le & Gascuel^59^ and the Gamma distribution. To estimate the reliability of the branches, 1000 boostraps were performed. Protein alignments were performed in Jalview v2.0^60^ using the TCoffee algorithm^61^ with defaults.

## Electronic supplementary material

Supplementary Information

Supplementary Table S4

Supplementary Table S5

Supplementary Table S7

## References

[1] Quinby, J. *Sorghum Improvement and the Genetics of Growth*. (Texas A&M University Press, 1974).

[2] Mullet J (2014). Energy sorghum-A genetic model for the design of C4 grass bioenergy crops. J. Exp. Bot..

[3] Slewinski TL (2012). Non-structural carbohydrate partitioning in grass stems: A target to increase yield stability, stress tolerance, and biofuel production. J. Exp. Bot..

[4] Murray SC (2008). Genetic improvement of sorghum as a biofuel feedstock: I. QTL for stem sugar and grain nonstructural carbohydrates. Crop Sci..

[5] McKinley B, Rooney W, Wilkerson C, Mullet J (2016). Dynamics of biomass partitioning, stem gene expression, cell wall biosynthesis, and sucrose accumulation during development of *Sorghum bicolor*. Plant J..

[6] Olson, S. N. *et al*. High biomass yield energy sorghum: Developing a genetic model for C4 grass bioenergy crops. *Biofuels*, *Bioprod*. *Biorefining* 640–655 (2012).

[7] Quinby JR, Karper RE (1954). Inheritance of height in sorghum. Agron. J..

[8] Graham D, Lessman KJ (1966). Effect of height on yield and yield components of two isogenic lines of *Sorghum vulgare* Pers. Crop Sci..

[9] Pereira MG, Lee M (1995). Identification of genomic regions affecting plant height in sorghum and maize. Theor. Appl. Genet..

[10] Casady AJ (1965). Effect of a single height (*Dw3*) gene of sorghum on grain yield, grain yield components, and test weight. Crop Sci..

[11] Truong SK, McCormick RF, Rooney WL, Mullet JE (2015). Harnessing genetic variation in leaf angle to increase productivity of *Sorghum bicolor*. Genetics.

[12] Multani DS (2003). Loss of an MDR transporter in compact stalks of maize *br2* and sorghum *dw3* mutants. Science.

[13] Monna L (2002). Positional cloning of rice semidwarfing gene, sd-1: Rice “Green Revolution gene” encodes a mutant enzyme envolved in gibberellin synthesis. DNA Res..

[14] Peng J (1999). “Green Revolution” genes encode mutant gibberellin response modulators. Nature.

[15] Brown PJ, Rooney WL, Franks C, Kresovich S (2008). Efficient mapping of plant height quantitative trait loci in a sorghum association population with introgressed dwarfing genes. Genetics.

[16] Hilley J, Truong S, Olson S, Morishige D, Mullet J (2016). Identification of *Dw1*, a regulator of sorghum stem internode length. PLoS One.

[17] Yamaguchi M (2016). Sorghum *Dw1*, an agronomically important gene for lodging resistance, encodes a novel protein involved in cell proliferation. Sci. Rep..

[18] Murphy RL (2011). Coincident light and clock regulation of pseudoresponse regulator protein 37 (PRR37) controls photoperiodic flowering in sorghum. Proc. Natl. Acad. Sci..

[19] Klein RR (2008). The effect of tropical sorghum conversion and inbred development on genome diversity as revealed by high-resolution genotyping. Crop Sci..

[20] Morishige DT (2013). Digital genotyping of sorghum-A diverse plant species with a large repeat-rich genome. BMC Genomics.

[21] Morris GP (2013). Population genomic and genome-wide association studies of agroclimatic traits in sorghum. Proc. Natl. Acad. Sci. USA.

[22] Higgins RH, Thurber CS, Assaranurak I, Brown PJ (2014). Multiparental mapping of plant height and flowering time QTL in partially isogenic sorghum families. G3 Genes, Genomes, Genet..

[23] Li X, Li X, Fridman E, Tesso TT, Yu J (2015). Dissecting repulsion linkage in the dwarfing gene *Dw3* region for sorghum plant height provides insights into heterosis. Proc. Natl. Acad. Sci.

[24] Thurber CS, Ma JM, Higgins RH, Brown PJ (2013). Retrospective genomic analysis of sorghum adaptation to temperate-zone grain production. Genome Biol..

[25] Quinby JR (1975). The genetics of sorghum improvement. J. Hered..

[26] Evans J (2013). Extensive variation in the density and distribution of DNA polymorphism in sorghum genomes. PLoS One.

[27] Ng PC, Henikoff S (2001). Predicting deleterious amino acid substitutions. Genome Res..

[28] Day IS, Miller C, Golovkin M, Reddy ASN (2000). Interaction of a kinesin-like calmodulin-binding protein with a protein kinase. J. Biol. Chem..

[29] Bögre L, Okrész L, Henriques R, Anthony RG (2003). Growth signalling pathways in Arabidopsis and the AGC protein kinases. Trends Plant Sci..

[30] Zegzouti H (2006). Structural and functional insights into the regulation of Arabidopsis AGC VIIIa kinases. J. Biol. Chem..

[31] Galván-Ampudia CS, Offringa R (2007). Plant evolution: AGC kinases tell the auxin tale. Trends Plant Sci..

[32] Rademacher EH, Offringa R (2012). Evolutionary adaptations of plant AGC kinases: From light signaling to cell polarity regulation. Front. Plant Sci..

[33] Yang S, Weers BD, Morishige DT, Mullet JE (2014). CONSTANS is a photoperiod regulated activator of flowering in sorghum. BMC Plant Biol..

[34] Zhang Y, McCormick S (2009). AGCVIII kinases: At the crossroads of cellular signaling. Trends Plant Sci..

[35] Barbosa ICR, Schwechheimer C (2014). Dynamic control of auxin transport-dependent growth by AGCVIII protein kinases. Curr. Opin. Plant Biol..

[36] Humphrey TV (2015). PERK-KIPK-KCBP signalling negatively regulates root growth in Arabidopsis thaliana. J. Exp. Bot..

[37] Tian J (2015). Orchestration of microtubules and the actin cytoskeleton in trichome cell shape determination by a plant-unique kinesin. Elife.

[38] Jonsson E, Yamada M, Vale RD, Goshima G (2015). Clustering of a kinesin-14 motor enables processive retrograde microtubule-based transport in plants. Nat. Plants.

[39] Vinogradova MV, Malanina GG, Reddy ASN, Fletterick RJ (2009). Structure of the complex of a mitotic kinesin with its calcium binding regulator. Proc. Natl. Acad. Sci..

[40] Vinogradova MV, Malanina GG, Waitzman JS, Rice SE, Fletterick RJ (2013). Plant kinesin-like calmodulin binding protein employs its regulatory domain for dimerization. PLoS One.

[41] Borassi C (2016). An update on cell surface proteins containing extensin-motifs. J. Exp. Bot..

[42] Bai L (2009). Plasma membrane-associated proline-rich extensin-like receptor kinase 4, a novel regulator of Ca2+ signalling, is required for abscisic acid responses in *Arabidopsis thaliana*. Plant J..

[43] Noh B, Murphy AS, Spalding EP (2001). Multidrug resistance-like genes of Arabidpsis required for auxin transport and auxin-mediated development. Plant Cell.

[44] Knöller AS, Blakeslee JJ, Richards EL, Peer WA, Murphy AS (2010). Brachytic2/ZmABCB1 functions in IAA export from intercalary meristems. J. Exp. Bot..

[45] Burow GB (2011). Registration of the BTx623/IS3620c recombinant inbred mapping population of sorghum. J. Plant Regist..

[46] Truong SK, McCormick RF, Morishige DT, Mullet JE (2014). Resolution of genetic map expansion caused by excess heterozygosity in plant recombinant inbred populations. G3 Genes, Genomes, Genet..

[47] Li H, Durbin R (2009). Fast and accurate short read alignment with Burrows-Wheeler transform. Bioinformatics.

[48] McKenna A (2010). The Genome Analysis Toolkit: A MapReduce framework for analyzing next-generation DNA sequencing data. Genome Res..

[49] DePristo MA (2011). A framework for variation discovery and genotyping using next-generation DNA sequencing data. Nat. Genet..

[50] van der Auwera, G. *et al*. From FastQ data to high-confidence variant calls: The Genome Analysis Toolkit best practices pipeline. *Curr*. *Protoc*. *Bioinforma*. **43**, 11.10.1–11.10.33 (2013). 10.1002/0471250953.bi1110s43 PMC4243306 25431634

[51] McCormick RF, Truong SK, Mullet JE (2015). RIG: Recalibration and interrelation of genomic sequence data with the GATK. G3 Genes, Genomes, Genet..

[52] Broman KW, Wu H, Sen Ś, Churchill GA (2003). R/qtl: QTL mapping in experimental crosses. Bioinformatics.

[53] Peng B, Yu RK, Dehoff KL, Amos CI (2007). Normalizing a large number of quantitative traits using empirical normal quantile transformation. BMC Proc..

[54] Manichaikul A, Moon JY, Sen Ś, Yandell BS, Broman KW (2009). A model selection approach for the identification of quantitative trait loci in experimental crosses, allowing epistasis. Genetics.

[55] Kwak IY, Moore CR, Spalding EP, Broman KW (2014). A simple regression-based method to map quantitative trait loci underlying function-valued phenotypes. Genetics.

[56] Tamura K, Stecher G, Peterson D, Filipski A, Kumar S (2013). MEGA6: Molecular evolutionary genetics analysis version 6.0. Mol. Biol. Evol..

[57] Edgar RC (2004). MUSCLE: A multiple sequence alignment method with reduced time and space complexity. BMC Bioinformatics.

[58] Edgar RC (2004). MUSCLE: Multiple sequence alignment with high accuracy and high throughput. Nucleic Acid Res..

[59] Le SQ, Gascuel O (2008). An improved general amino acid replacement matrix. Mol. Biol. Evol..

[60] Waterhouse AM, Procter JB, Martin DMA, Clamp M, Barton GJ (2009). Jalview Version 2-A multiple sequence alignment editor and analysis workbench. Bioinformatics.

[61] Notredame C, Higgins DG, Heringa J (2000). T-Coffee: A novel method for fast and accurate multiple sequence alignment. J. Mol. Biol..

