# Supplementary material for: Sorghum Dw2 Encodes a Protein Kinase Regulator of Stem Internode Length
Source: Sci Rep. 2017 Jul 4;7:4616. doi: 10.1038/s41598-017-04609-5 (PMC5496852; doi:10.1038/s41598-017-04609-5)
Supplement: Supplementary file 1 — Supplementary Information [file 41598_2017_4609_MOESM1_ESM.pdf]

Supplementary Information

Sorghum *Dw2* Encodes a Protein Kinase Regulator of Stem Internode Length

Josie L. Hilley[1], Brock D. Weers[2], Sandra K. Truong[1], Ryan F. McCormick[1], Ashley J. Mattison[1], Brian A. McKinley[2], Daryl T. Morishige[2], and John E. Mullet[1,2*]

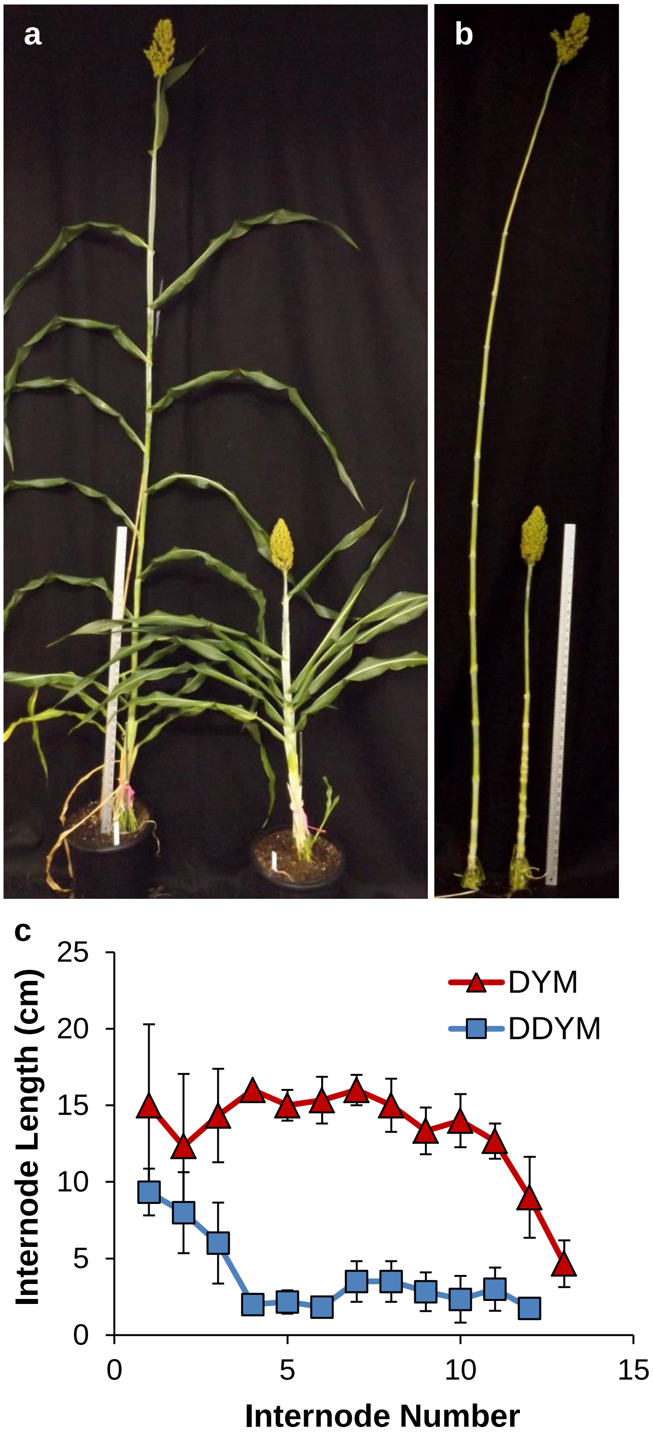

Supplementary Figure S1. Length of each internode for Dwarf and Double Dwarf Yellow Milo. DYM and DDYM (n=3 per line) were grown in the greenhouse in the fall and harvested at anthesis. Plants are shown with leaves (**a**) and without (**b**). For each photograph, DYM is on the left and a meter stick is shown for reference (**a** and **b**). The plants were measured for the length of each internode, numbered from the peduncle, with the average and standard deviation for each internode shown (**c**).

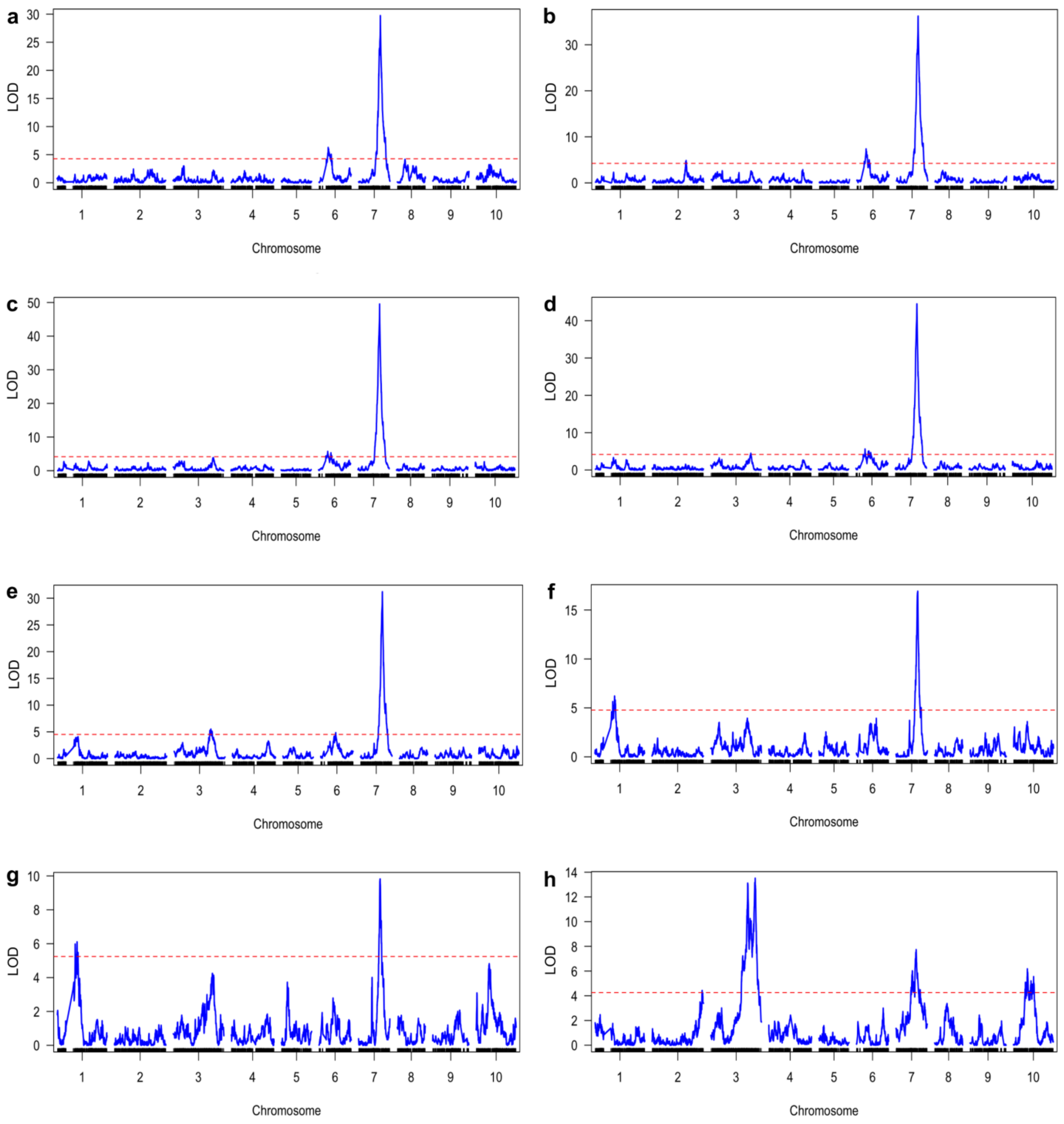

Supplementary Figure S2. Internode length QTL identified using BTx623 x IS3620c RILs. The RIL population was grown in the greenhouse and genotyped using DG. QTL mapping was performed in R/qtl using IM. For each graph, the markers from the genetic map are listed on the x-axis and the LOD score on the y-axis. Each graph is for a different internode starting with the second internode below the peduncle (**a**) and ending with the eighth internode below the peduncle (**g**). The last graph is the peduncle (**h**).

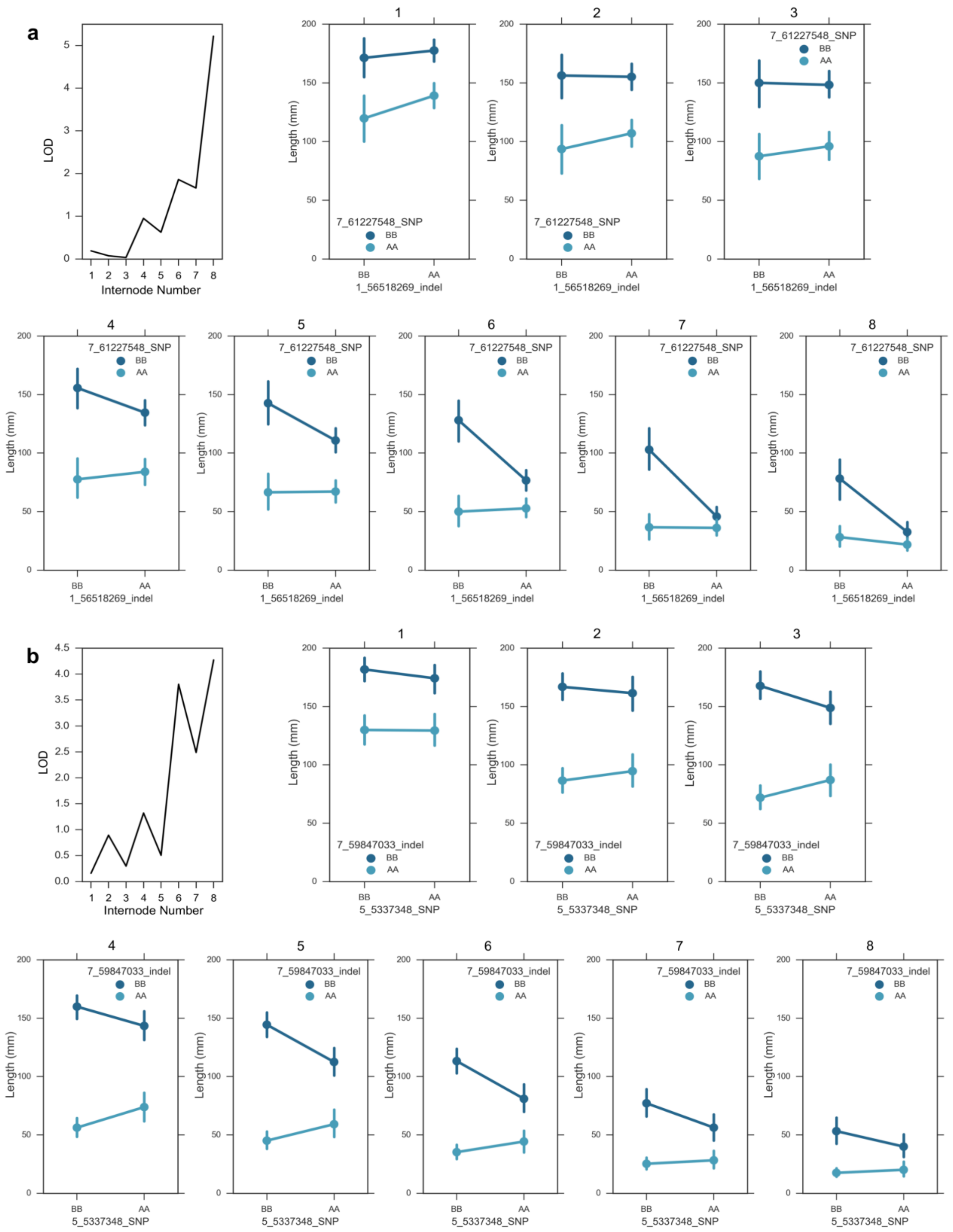
a
LOD
Internode Number
1
2
3
4
5
6
7
8
Length (mm)
7_61227548_SNP
BB
AA
1_56518269_indel
b
LOD
Internode Number
Length (mm)
7_59847033_indel
BB
AA
5_5337348_SNP

c

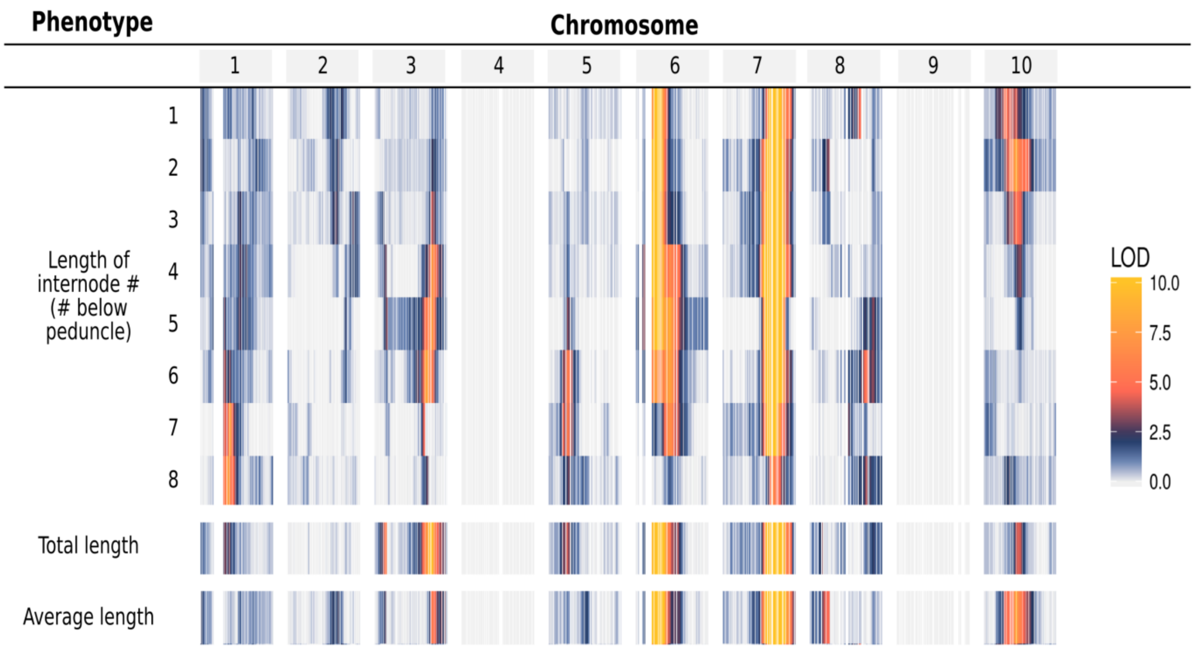

Supplementary Figure S3. Multiple QTL mapping (MQM) in BTx623 x IS3620c RILs. For all, MQM was performed on the same genotype and phenotype data as IM. MQM was performed in R/qtl . IM was used to seed multiple QTL model selection for each trait. The best model for each trait was combined to form the composite multiple-QTL model. This model consists of 11 QTL and two epistatic interactions. (**a**) and (**b**) a graph of the LOD score for the epistatic interaction for each internode length trait . The following eight graphs are the phenotype (y-axis) for each combination of genotype (x-axis and series) for each internode length (1-8). (**a**) is the interaction between a QTL on chromosome 1 and a QTL near *Dw3* while (**b**) is the interaction between a QTL on chromosome 5 and *Dw3*. (c) A heat map of the LOD value for each DG marker for each trait based on composite MQM analysis.

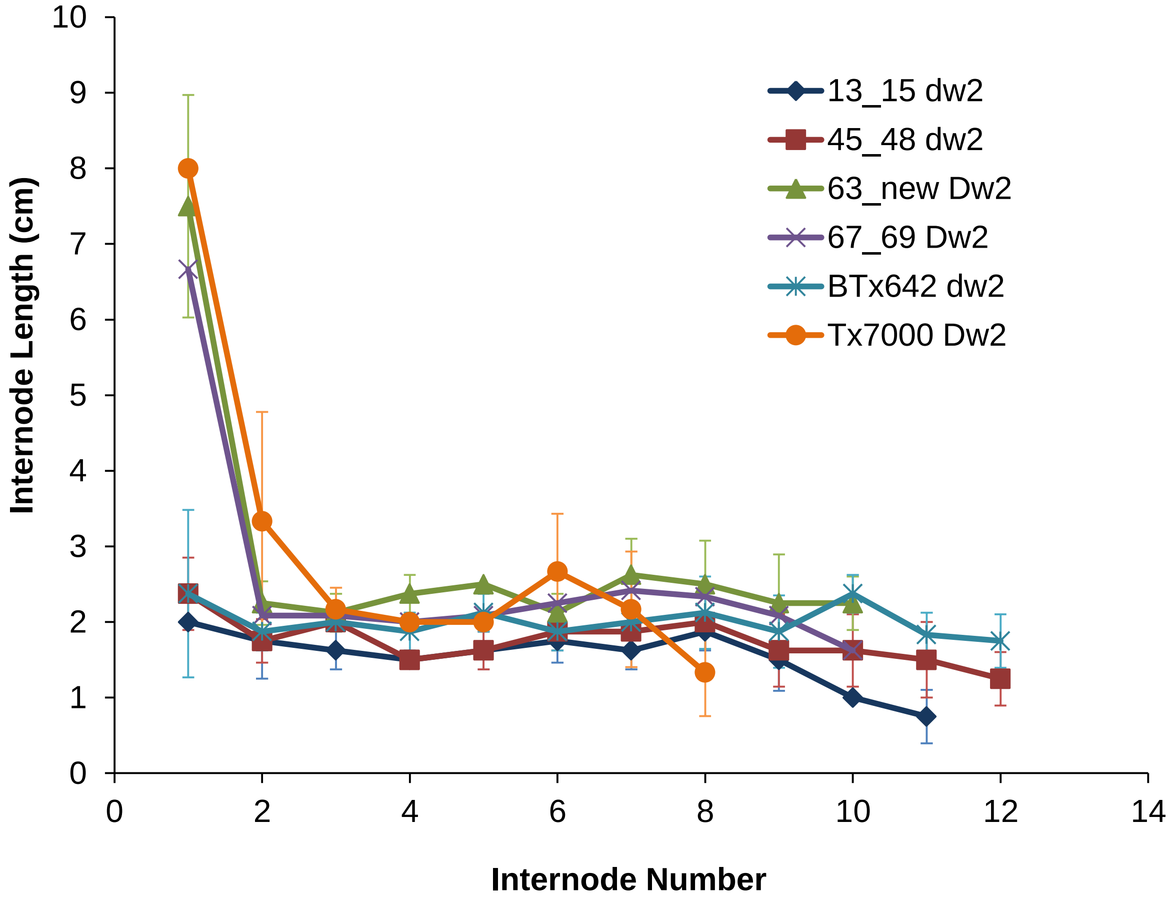

Supplementary Figure S4. Internode length phenotypes for select BTx642 x Tx7000 RILs. The RILs (n=4 per line) are the lines that had a close breakpoint in the *Dw2* delimited region. At grain maturity, the length of each internode was measured with the average and standard deviation shown. These lines were grown in the winter under low light intensity in two different greenhouses. Only one greenhouse set is shown, though both are similar.

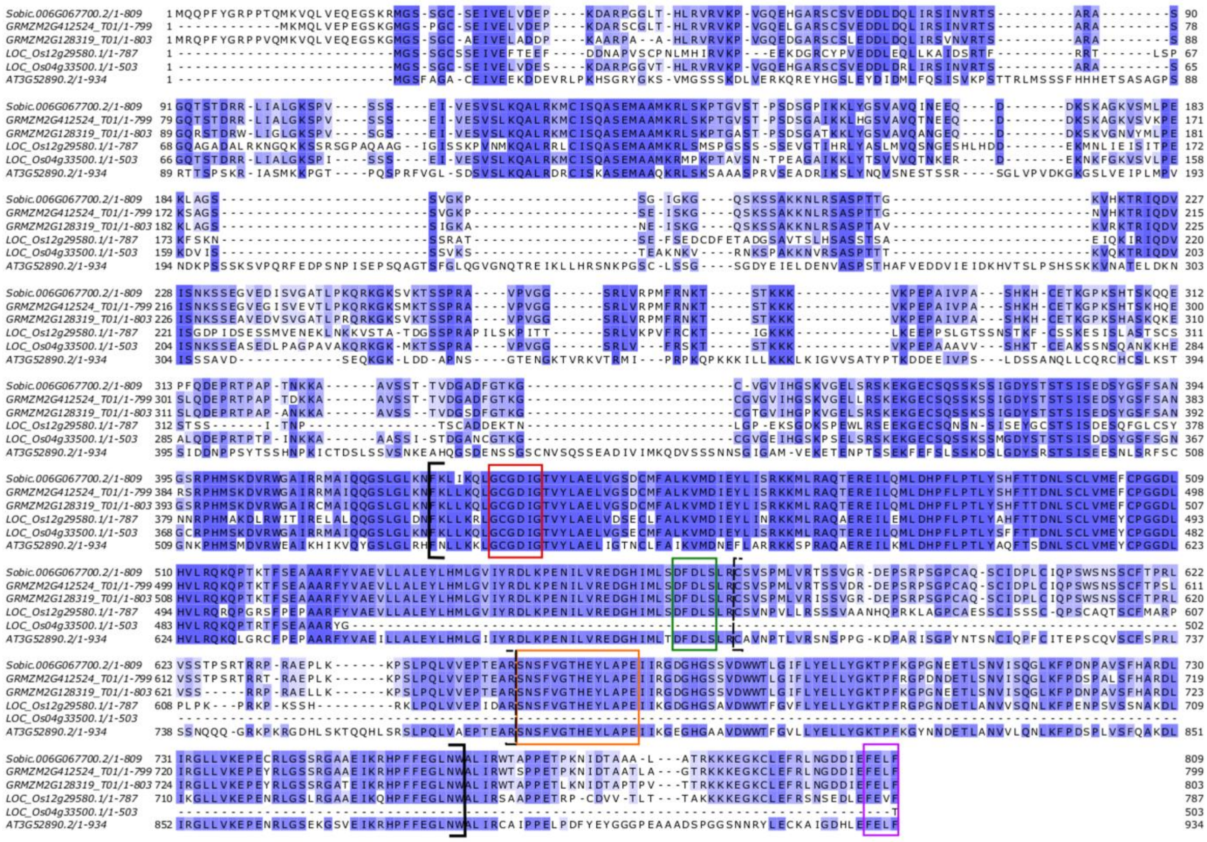

Supplementary Figure S5. Alignment of sorghum Dw2 with the maize, rice, and Arabidopsis homologs. A multiple sequence alignment was made in Jalview 2.0 with TCoffee between Dw2 (Sobic.006G067700), two closest maize homologs (GRMZM2G412524 & GRMZM2G128319), two closest rice homologs (LOC_Os12g29580 & LOC_Os04g33500), and the closest Arabidopsis homolog, KIPK (AT3G52890). The kinase domain is demarcated with solid brackets. Dashed brackets demarcate the insertion domain. A red box labels the highly conserved GxGxxG sequence that functions in ATP binding. A green box labels the DFDLS Mg^{2+} binding site and the orange box denotes the T-loop that is phosphorylated to activate the kinase. Finally, the purple box highlights the PDK-interacting fragment.

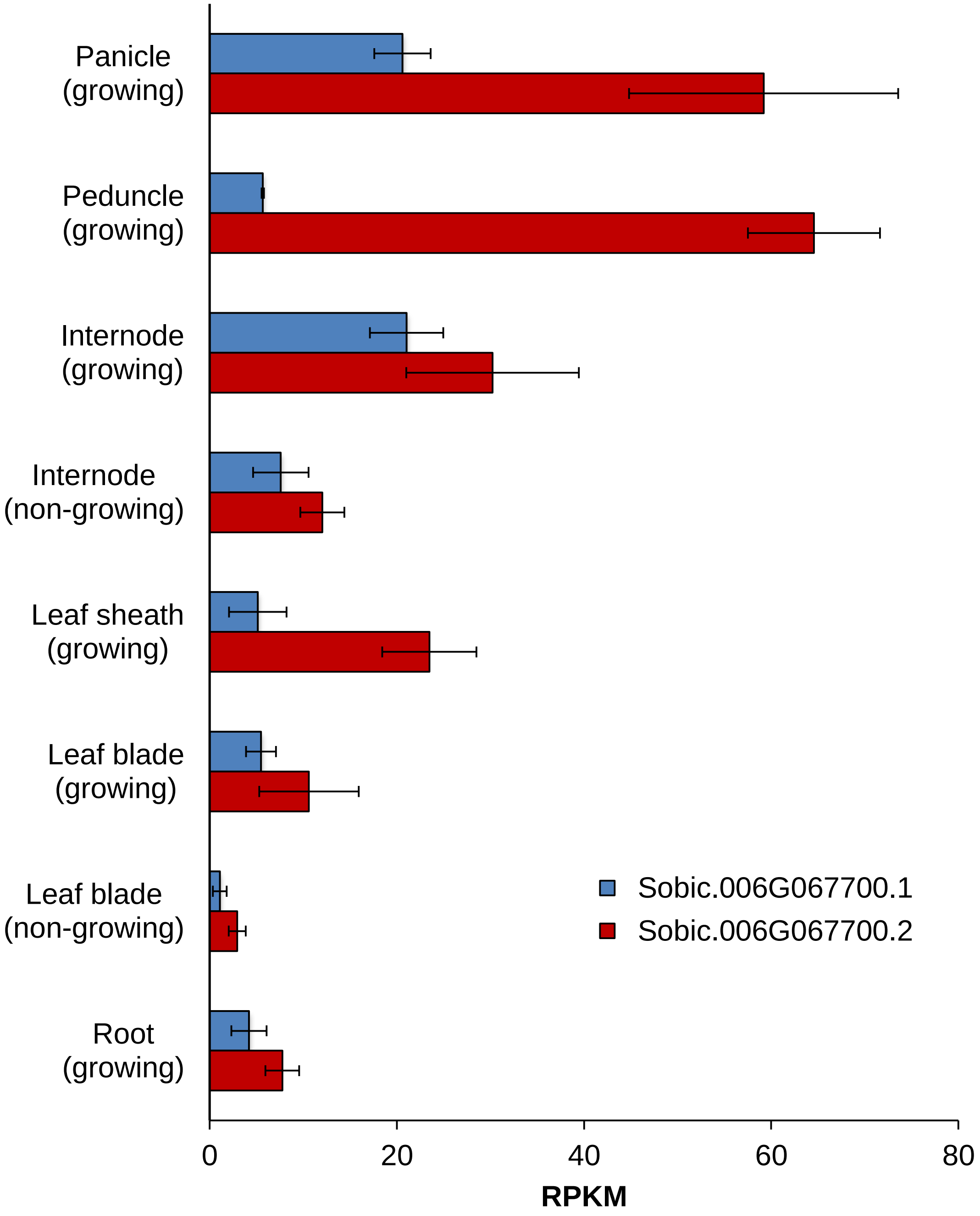

Supplementary Figure S6. Expression of *Dw2* in various tissues. RNA was collected from the various tissues at 44 DAE (n=3). RNA was used for RNA-seq performed by the DOE Joint Genome Institute (Phytozome). The RNA-seq data was analyzed as described in McKinley et al[5]. The average RPKM is shown with the standard error of the mean. Both transcripts are shown with Sobic.006G067700.2 being the primary transcript.

Supplementary Table S1. Summary of the Best Model from MQM of Individual Phenotypes. Chromosome is shortened to "Chr."

Trait	QTL	Chr	Peak LOD	Peak (cM)	Peak (bp)	Start	Stop	Interactions (with Number; LOD)
Total Length	1	3	6.4	29.78	4309508	3057129	6163945	
	2	3	10.73	136.63	67760473	64467623	68260513	
	3	6	14.19	30.67	42508419	41934840	43596665	
	4	7	33.46	73.76	59847033	59247435	59991087	
	5	10	3.5	61.19	9626445	7791830	52293650	
Average Internode Length	1	2	3.18	120.91	66477452	61525510	69131669	
	2	3	5.35	136.63	67760473	65905794	72466480	
	3	6	18.33	31.71	42785280	41934840	43596665	
	4	7	42.44	73.76	59847033	59504276	59991087	
	5	8	4.03	28.3	3669596	2194037	53066186	
	6	10	7.39	60.06	9375593	8197931	11829372	
Internode 1 Length	1	6	15.59	31.71	42785280	42085051	43596665	
	2	7	16.56	73.76	59847033	59504276	61227548	
	3	10	4.22	38.41	5551100	4709177	51917685	
Internode 2 Length	1	6	9.72	29.36	42085051	39890464	43596665	
	2	7	25.15	73.76	59847033	59504276	59991087	
	3	10	5.96	60.06	9375593	6931729	54111672	
Internode 3 Length	1	3	4.57	135.53	67047035	64467623	69688959	
	2	6	14.55	29.36	42085051	41934840	43596665	
	3	7	30.09	71.36	59504276	59051589	59991087	
	4	10	4.71	60.06	9375593	6931729	51917685	
Internode 4 Length	1	3	7.11	135.53	67047035	65905794	69174377	
	2	6	11.29	29.36	42085051	39890464	43596665	
	3	6	3.37	64.34	51939240	49361779	53985519	
	4	7	3.98	69.75	59247435	58919267	59847033	
	5	7	5.7	73.76	59847033	59504276	59991087	
	6	10	2.94	69.97	47978440	1707726	55494359	
Internode 5 Length	1	3	7.08	135.53	67047035	63288516	68260513	
	2	6	10.02	29.36	42085051	39890464	45706034	
	3	7	36.41	71.36	59504276	59247435	59847033	
Internode 6 Length	1	1	5.21	56.53	21177180	8262098	57277940	
	2	3	8.95	120.25	61770650	60898775	63875751	
	3	6	6.54	29.36	42085051	1659623	50325848	
	4	7	26.94	71.36	59504276	59051589	59847033	
	5	8	3.88	75.53	59711692	58297740	61022028	
Internode 7 Length	1	1	10.32	66.73	56518269	54249162	58058247	6; 3.530
	2	3	6.54	120.25	61770650	60175252	63875751	
	3	5	7.29	27.92	5337348	3504889	6175733	5; 2.713
	4	6	8.07	51.47	48844243	46697460	50423659	
	5	7	13.6	71.36	59504276	59051589	59991087	3; 2.713
	6	7	3.9	82.07	61227548	60996573	63995754	1; 3.530
Internode 8 Length	1	1	6.29	65.71	55803782	8262098	58058247	
	2	7	6.04	73.76	59847033	59247435	60577582	
	3	8	4.46	75.53	59711692	57593772	62528965	

Supplementary Table S2. Summary of the Best Model for Each Trait Based on Composite MQM.
Includes the two interactions, notated with an "&". Chromosome is shortened to "Chr" and internode is shortened to "Int". * P < 0.05, ** P < 0.01, *** P < 0.001.

				LOD									
**QTL**	**Chr**	**Location (cM)**	**Location (Mb)**	**Total Length**	**Average Int Length**	**Length Int 1**	**Length Int 2**	**Length Int 3**	**Length Int 4**	**Length Int 5**	**Length Int 6**	**Length Int 7**	**Length Int 8**
1	1	66.7	56.52	1.73**	0.29	0.75	0.21	0.06	0.07	0.73	2.49***	4.33***	5.69***
2	2	120.9	66.48	0.32	2.7***	1.31*	2.74***	1.49*	1.2*	0.22	0.35	0.35	0.04
3	3	29.8	4.31	6.27***	2.68***	0.99*	0.92*	1.9**	2.37**	2.79***	1.75**	1.66**	0.02
4	3	136.6	67.76	10.97***	5.65***	1.41*	2.24**	3.99***	6.24***	6.16***	4.55***	1.39*	1.83**
5	5	27.9	5.34	1.11*	0.09	0.03	0.08	0.02	0.02	1.34*	2.84***	2.83***	1.21*
6	6	31.7	42.79	15.91***	20.25***	17.73***	11.82***	15.09***	13.06***	10.42***	5.17***	1.78**	0.07
7	7	73.8	59.85	18.61***	20.5***	5.34***	13.38***	16.02***	24.05***	20.58***	11.21***	10.2***	3.64***
8	7	82.1	61.23	0.01	0.98*	1.2*	0.05	0.03	0.05	0.01	0	1.55**	0.09
9	8	28.3	3.67	2.7***	4.32***	0.73	3.18***	2.65***	1.31*	1.1*	0.14	0	0.04
10	8	75.5	59.71	1.15*	0.03	0.6	0	0.12	0.45	2**	3.35***	2.08**	3.44***
11	10	60.1	9.38	4.15***	7.97***	4.33***	7.01***	5.26***	2.01**	1.11*	0.07	0.2	1.17*
1&8	n.a.	n.a.	n.a.	0.01	0.2	0.19	0.07	0.03	0.95*	0.63	1.86**	1.66**	5.22***
5&7	n.a.	n.a.	n.a.	0.04	0.05	0.16	0.89*	0.3	1.32*	0.51	3.8***	2.49***	4.27***

Supplementary Table S3. Additional Notes on the Genes in the Delimited Region of *Dw2*.

Gene	Description	Maize Homolog(s)	Notes
Sobic.006G067000	PPR repeat	GRMZM2G163043	
Sobic.006G067050	Unknown	None	Low levels of gene expression
Sobic.006G067100	rRNA N-glycosylase	GRMZM2G013331 & GRMZM2G022095	Maize homologs lack the first part of the gene
Sobic.006G067150	Unknown	GRMZM2G017933	BLAST match has limited percent identity
Sobic.006G067200	Unknown	None	
Sobic.006G067300	F-box domain	GRMZM2G015349 & GRMZM2G125954 & GRMZM2G435096	BLAST matches have limited percent identity; very low levels of gene expression
Sobic.006G067400	Calcineurin-like phosphoesterase	GRMZM2G128399	
Sobic.006G067500	Calcineurin-like phosphoesterase	GRMZM2G128399	First ~220 residues lack homology to maize homolog
Sobic.006G067600	Histone deacetylase	GRMZM2G119703	
Sobic.006G067700	Ribosomal protein S6 kinase	GRMZM2G412524 & GRMZM2G128319	

Supplementary Table S6. Primers for Fine Mapping SNPs.

Name	Forward Primer	Reverse Primer	SNP Location
SNP_66800	CACTCATAGCTGAGGAGAAACC	TAACCAGGATGCCCAAACTC	42,710,479
SNP_66900	CTTCTTTCGAGACCTCCTTCATT	TCTGGTTATTGGCAGGAGATTAC	42,723,163
SNP_67000	CGCCGAATGCTGTTACCTATAA	GCCATAGCTTAGTTCCTCCTAAC	42,724,389
SNP_67050	CAACACTAAACACCAGCACAAC	GGCCAGGCTTCTAAATAGTAGAG	42,751,429
SNP_67700	TCGGTGGAGGATGATCTTGA	TTCCGAAACATTGGCCTCACCA	42,806,049
SNP_67800	ATGGTGACATGTGAGGTCTATTT	GTTACTGGACTGAAGAACCAGAG	42,822,513
